# IMCC: A Novel Quantitative Approach Revealing Variation of Global Modular Map and Local Inter-Module Coordination Among Differential Drug’s Targeted Cerebral Ischemic Networks

**DOI:** 10.3389/fphar.2021.637253

**Published:** 2021-04-16

**Authors:** Pengqian Wang, Yanan Yu, Jun Liu, Bing Li, Yingying Zhang, Dongfeng Li, Wenjuan Xu, Qiong Liu, Zhong Wang

**Affiliations:** ^1^ Institute of Chinese Materia Medica, China Academy of Chinese Medical Sciences, Beijing, China; ^2^ Institute of Basic Research in Clinical Medicine, China Academy of Chinese Medical Sciences, Beijing, China; ^3^ Institute of Information on Traditional Chinese Medicine, China Academy of Chinese Medical Sciences, Beijing, China; ^4^ Dongzhimen Hospital, Beijing University of Chinese Medicine, Beijing, China; ^5^ School of Mathematical Sciences, Peking University, Beijing, China; ^6^ School of Life Sciences, Beijing University of Chinese Medicine, Beijing, China

**Keywords:** IMCC, modular map rewiring, inter-module coordination, multiple-target drug, cerebral ischemia

## Abstract

Stroke is a common disease characterized by multiple genetic dysfunctions. In this complex disease, detecting the strength of inter-module coordination (genetic community interaction) and subsequent modular rewiring is essential to characterize the reactive biosystematic variation (biosystematic perturbation) brought by multiple-target drugs, whose effects are achieved by hitting on a series of targets (target profile) jointly. Here, a quantitative approach for inter-module coordination and its transition, named as IMCC, was developed. Applying IMCC to mouse cerebral ischemia–related gene microarray, we investigated a holistic view of modular map and its rewiring from ischemic stroke to drugs (baicalin, BA; ursodeoxycholic acid, UA; and jasminoidin, JA) perturbation states and locally identified the cooperative pathological module pair and its dissection. Our result suggested the global modular map in cerebral ischemia exhibited a characteristic “core–periphery” architecture, and this architecture was rewired by the effective drugs heterogeneously: BA and UA converged modules into an intensively connected integrity, whereas JA diverged partial modules and widened the remaining inter-module paths. Locally, the PMP dissociation brought by drugs contributed to the reversion of the pathological condition: the focus of the cellular function shift from survival after nervous system injury into development and repair, including neurotrophin regulation, hormone releasing, and chemokine signaling activation. The core targets and mechanisms were validated by *in vivo* experiments. Overall, our result highlights the holistic inter-module coordination rearrangement rather than a target or a single module that brings phenotype alteration. This strategy may lead to systematically explore detailed variation of inter-module pharmacological action mode of multiple-target drugs, which is the principal problem of module pharmacology for network-based drug discovery.

## Introduction

Stroke is a common disease characterized by multiple genetic dysfunctions (Matthew et al., 2012; Zhou et al., 2018). The discovery of the multi-target therapeutic drugs is considered as a potential solution for reversing the biomolecular network of disease systematically to achieve homeostasis (Frantz, 2005; Roth et al., 2004; Wang et al., 2015a). How to clarify the “shotguns-like” action mode of multi-target drugs is still far from clear. Network-based drug analysis aims to harness explosion of high-throughput data to investigate the pharmacology of drugs, which makes it feasible to understand the intrinsic pharmacological mechanism of multi-target drugs (Cheng et al., 2019). As accumulated data from high-throughput technologies delineate a holistic view of intracellular molecular network, the major challenge in the post-genomic era is deciphering how these entities in the cell work together to execute sophisticated functions (Wang and Wang, 2013; Kim et al., 2014). The ongoing efforts have been made in decomposing a network into modules and identifying the targeted modules of drugs (Wang et al., 2012). This may help to decipher modularized function organization in targeted networks and reveal the pharmacological mechanisms of multi-target drugs.

However, inter-module connections, as the “backbone” contributing to functional coordination and information flow between modules in most biological processes, are ever important (Granovetter, 1973; Lin et al., 2012; Ma and Gao, 2012; Onnela et al., 2009; Levy et al., 2017). The inter-module connections, which are more transient and flexible than intra-module connections (Ma and Gao, 2012; Kim et al., 2014), could be considered as targets of drugs, since modular rearrangement brought by inter-module relationship transitions may provide more efficient ways for phenotype alteration (Amar et al., 2013; Meda et al., 2014) than genetic variation or modular allostery (Roguev et al., 2008; Bandyopadhyay et al., 2010; Costanzo et al., 2016). Such modular rewiring of conserved functional modules can be used as a network biomarker to characterize the dynamics of drug responses (Zeng et al., 2014), by identifying and evaluating the drug-conditional existence of collaborations between modules (Zeng et al., 2013). Especially for the multiple-target drugs, it is an extremely interesting and promising perspective to apply inter-module connectivity analysis to reveal the mechanism of pharmacology (Wang et al., 2011; Ding et al., 2015; Liu and Wang, 2015). A set of studies have referred the quantitative evaluation method for inter-module connections (Ulitsky et al., 2008; Missiuro et al., 2009; Hsu et al., 2011; Kelder et al., 2011; David and Ron, 2014); for example, the number of interactions or overlapping nodes between modules or community is commonly considered as the connections between modules (Yang et al., 2009; Bandyopadhyay et al., 2010; Tesson, et al., 2010; Kelder et al., 2011; Amar et al., 2013). Many of these addressed the problem of module detection and module-to-module interactions simultaneously but did not treat inter-module assessment as a main task. Algorithms for inter-module assessment are still far from perfect. Furthermore, it is pertinent to introduce biological function to measure reliability and validity of inter-module evaluations. And how to quantify transition of inter-module coordination related to pharmacological mechanisms remains unknown.

Baicalin (BA), ursodeoxycholic acid (UA), and jasminoidin (JA) are three major components contained in Qingkailing injection, an effective preparation widely prescribed to patients with ischemic stroke. Our previous studies showed that each of BA, UA, and JA significantly reduced the infarction volume in the ischemic brain and exerts neuroprotective effects by inhibiting inflammatory response in cerebral ischemia (Liu et al., 2012; Wang et al., 2015b; Wang et al., 2018). These studies provide “targeting section” of these multi-target drugs. Nevertheless, it is still unclear to characterize the overview of functional module rewiring response to distinct drug perturbations. The underlying mechanisms of these drugs regarding the inter-module coordination in modulating complex disease phenotypes are still to be explored.

**FIGURE 1:**
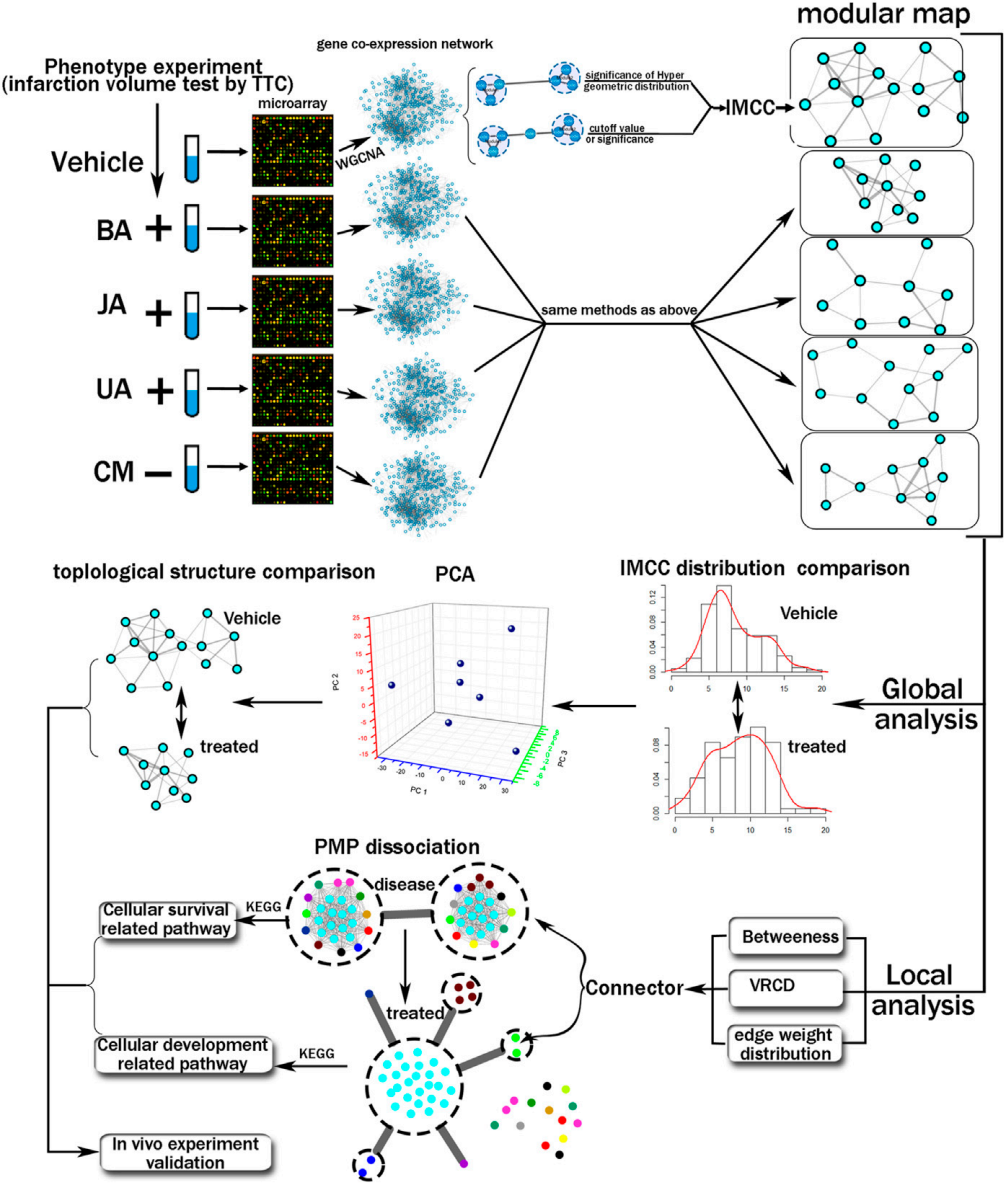
Schematic diagram of the systematic strategies used to reveal variation of global modular map and local inter-module coordination.

In this study, we propose an integrated computational and experimental approach to the systematic discovery of differential transition of inter-module coordination that are causal determinants of phenotype alteration (Figure 1). Here, the gene expression profile of hippocampus from MCAO (middle cerebral artery obstruction) mice treated by BA, JA, and UA was analyzed by cDNA microarray, and the weighted gene co-expression network and modules are constructed accordingly. First, we integrated the quantitative methods and statistical analysis to construct an inter-module coordination coefficient (IMCC) to evaluate the module-to-module cooperation. Next, the inter-module coordination rewiring across treated condition to disease was evaluated and compared globally. Third, the most closely coordinating module pair was further analyzed using the dissection rate and KEGG pathway. Finally, the mechanism of BA, UA, and JA was validated by *in vivo* experiments.

## Materials and Methods

### Animals Model and Drug Administration

All animal experiments conducted were approved by the Ethics Committee of China Academy of Chinese Medicine. The operation was performed according to NIH Guidelines for the Care and Use of Laboratory Animals for Experimental Procedures (National Research Council, 1996). A total of 126 mice were used to pharmacodynamic experiment and transcriptome data examination in this study. Animals were randomized into sham-operated, vehicle, BA-treated, JA-treated, UA-treated, and CM-treated groups. Except the sham-operated group, all animals were operated for middle cerebral artery obstruction to induce focal cerebral ischemia–reperfusion model. Briefly, the left middle cerebral artery was occluded using an intraluminal filament. The artery was ligated for 1.5 h, after that reperfused for about 24 h. For animals in the sham group, the external carotid artery (ECA) was sham-operated and prepared surgically without filament inserting. Drugs were injected into the tail vein for 2 ml/kg immediately after modeling. The concentration of drugs was BA in 5 mg/ml, JA in 25 mg/ml, UA in 7 mg/ml, and CM (concha margaritifera) in 50 mg/ml.

### Drug Efficacy Examination and Transcriptome Data Analysis

For each group, nine mice were used to examine infarction volume by TTC (2, 3, 5-triphenyltetrazolium chloride staining). The volume of the infarct region was determined by Pathology Image Analysis System (Topica Inc.) and was recognized to reflect efficacy of drugs.

After reperfusion for 24 h, the hippocampus of 12 animals of each group was sliced and homogenized using TRIzol reagent, and total RNA was extracted and purified. Then, cDNA microarray consisted of a collection of 374 ischemia-related genes, including 114 genes related to stroke and 260 genes in pathways related to cerebral ischemia. Expression data of the 374 genes were uploaded to the public database ArrayExpress (http://www.ebi.ac.uk/arrayexpress/, E-TABM-662).

### Co-Expression Network Construction and Module Detection

Based on the expression of aforementioned 374 genes, co-expression network was constructed and modules were identified using weighted gene co-expression network analysis (WGCNA) R package (Langfelder and Horvath, 2008). WGCNA can be used for finding clusters (modules) of highly correlated genes, for summarizing such clusters for relating modules to one another (Langfelder and Horvath, 2008). Correlation networks facilitate network-based gene screening methods that can be used to identify candidate therapeutic targets or further inter-module analysis (Langfelder and Horvath, 2008). Correlation network in different conditions was related to genes’ expression pattern. In this study, we aim to calculate inter-module coordination, which may reflect the disease- and drug-conditional existence of collaborations between modules, to facilitate the further pharmacological investigation. Therefore, we employed the WGCNA to construct weighted gene co-expression network.

In brief, the weighted network was fully specified by its adjacency matrix, which was constructed between all pairs of probes across the measured samples by using appropriate adjacency function parameters (β) for each group (β = 8 for vehicle, seven for CM, four for BA, 12 for JA, and eight for UA) (Langfelder and Horvath, 2008; Li et al., 2016). The soft thresholds were selected when the network gets the best scale-free topology criterion. As a result, adjacency functions a_ij_ ≥ 0.02 were used to construct weight gene co-expression network and further analysis.

The WGCNA identified gene modules using average linkage hierarchical clustering with topologic overlap measure and Dynamic Hybrid Tree Cut algorithm (Langfelder and Horvath, 2008); modules are subsequently assigned a color as names, and the details of the module detection were shown in our previous study (Li et al., 2016).

### Inter-Module Coordination Analysis

According to the local structure, connections between two modules composed of edges between nodes from distinct modules were defined as direct inter-module connections (DIMC), and interactions mediated by genes that associate with both the two modules were classified as indirect inter-module connections (IIMC). We firstly calculated two types of correlation parameters: SW for DIMC; CT and PS for IIMC; then, we screened these parameters using hypergeometric distribution or cutoff value and integrated the identified parameters; finally, we optimized the integration weight according to KEGG database.

### Parameters Calculations

For SW, we calculated the sum of weight of edges between pairs of modules:

$$SW\left(Mx,My\right)=\sum_{i\in Mx,j\in My}aij,$$ (1)

where *M*_*x*_ and *M*_*y*_ denote any two modules connected by at least one edge, *i* and *j* are a gene in *M*_*x*_ and *M*_*y*_, respectively, and *a*_*ij*_ is the weight of edge between gene *i* and *j*. Using this formula, we calculated the direct inter-module connections for any module pair possessing one or more edges.

To decide whether the inter-module direct connections were statistically significant, we used the *p* value of the hypergeometric distribution (Lin et al., 2012), defined as

$$p=\sum_{k=x}^{n}\frac{\left(\begin{array}{c} M\\ k \end{array}\right)\left(\begin{array}{c} N-M\\ n-k \end{array}\right)}{\left(\begin{array}{c} N\\ n \end{array}\right)},$$ (2)

where *x* is observed inter-module connections; *k* and *n* are the numbers of inter-module connections and all possible edges between two modules, respectively; and *M* and *N* represent the total numbers of inter-module connections and all combinational gene pairs between any two modules in a module-to-module network, respectively. If *p* < 0.05, the *SW* is defined as a valid direct measurement.

For indirect inter-module connections, we introduced two parameters: path strength (*PS*) and consistency score (*CT*).

In the light of the network, paths consisted of multiple vertexes and links between them (Kelder et al., 2011). To simplify the problem, we restricted the length of paths and only considered paths that consist of three nodes [outset (*o*), mediation (*m*), and end (*e*)] with two links.

$$PS\left(Mx,My\right)=\sum_{\begin{array}{l} o\in Mx,e\in My\\ m\notin Mx,m\notin My \end{array}}\frac{Wm,o}{Wm}•\frac{Wm,e}{Wm}.$$ (3)

The path strength (*PS*) of a path is defined as the product of the weighted probabilities that mediation chooses outset and end. The weighted probability from *m* to *o* is the ratio of the weight between *m* and *o* (*W*_*m*_ *,o*) to the sum of the weights between *m* (*W*_*m*_) and its first neighbors, the same as *m* to *e*.

Hypergeometric distribution was also used to screen the statistically significant *PS*. However, different from *SW*, in Eq. 2, *x* is observed nodes connecting a pair of module; *k* and *n* are the numbers of nodes connecting a pair of modules and all possible nodes connecting the two modules, respectively; and *M* and *N* represent the total numbers of nodes connecting any pair of module and all possible nodes between any two modules in a module to module network, respectively.

We also employed consistency score (*CT*) to measure the inter-module connectivity as described in (Hsu et al., 2011).

$$CTscoreM_{x},M_{y}=\sum_{i\in G}\left(Min\left\{\left(C_{M_{x}},i-\frac{S}{C}CLi\right),\left(C_{M_{y}},i-\frac{T}{C}CLi\right)\right\}\times \frac{C_{M_{x}},i\times C_{M_{y}},i}{CLi}\times Wi\right).$$ (4)

*G* is a gene set that consists of all genes in network, and *C* is the total number of genes in *G*. *CL*_*i*_ is the total number of links to gene *i*; *W*_*i*_ is the weight of gene *i* in network. *S* and *T* are the numbers of genes in modules *M*_*x*_ and *M*_*y*_, respectively; *CM*_*x,i*_ and *CM*_*y,i*_ are the observed numbers of links connecting gene *i* and modules *M*_*x*_ and *M*_*y*_, respectively. Eq. 4 was used to compare the weights of genes correlated with a pair of modules with the weights of genes related to only one of the modules (Hsu et al., 2011). As the *CT* is a value after comparison with theoretical value, we set cutoff value (10) to screen out the valid *CT*.

### Measurement Integration

To obtain a more accurate and objective relationship between modules, we merged *DIMC* and *IIMC*. First, we set *SW* of the inter-module connections, whose *p*-value of hypergeometric distribution was less than 0.05, as weight of *DIMC*. In the process of *IMCC*_*1*_ integration, *SW* and *CT* were two parameters of different dimensions, so it was adopted to correct the two parameters to the value of 0–1 (Supplementary Table S1). We normalized the two measurements to be numbers between [0–1] by the follow formula:

$${f^{\prime }}_{x}=\frac{f_{x}-f_{\min}}{f_{\max}-f_{\min}}.$$ (5)

The two parameters were weighted using weighted coefficients α and β for *SW* and *CT*, respectively. The two weighted parameters were summed up, as follows:

$$IMCC_{1}=\alpha •SW+\beta •CT.$$ (6)

We set α + β = 1 and coefficient ratio ρ = α/β. By adjusting *ρ* value, the effect of *SW* and *CT* on *IMCC*_1_ would be altered. We calculated the *IMCC*_*1*_ when ρ = 1/10, 1/8, 1/4, 1/2, 1/1, 2/1, 4/1, 8/1, and 10/1, respectively.

In the integration of *SW* and *PS*, both of them belonged to the same dimension, so we plus the two parameters without weighting (Supplementary Table S2), defined as:

$$IMCC_{2}=SW+PS.$$ (7)

### Optimization and Verification of Weighting Coefficient

In biological networks, the communication between certain modules is commonly mediated by component with important functions; for example, a gene might be a target regulated by two modules competitively. All the inter-module correlations summarized or predicted are presumed to contribute to biological functions. Therefore, it is imperative to introduce biological data to define the best weighting coefficient, in order to select the optimal *IMCC*. As a result, we employed the KEGG (Kyoto Encyclopedia of Genes and Genomes) database, according to which we calculated the Jaccard similarity of enriched pathways of each module pair.

$$JS=\frac{\left|A\cap B\right|}{\left|A\cup B\right|}.$$ (8)

*A* and *B* is the category of enriched KEGG terms in module *M*_*x*_ and *M*_*y*_, respectively. Therefore, *A* ∩ *B* represents the number of identical categories of KEGG terms between *A* and *B*, and *A* ∪ *B* represents the number of all the categories of KEGG terms in both A and B. For example, if *A* ∩ *B* = 6 and *A* ∪ *B* = 11, then *JS* will be 0.54545. We presumed that modules enriched with the same KEGG categories might form more dense connections than those with different KEGG categories. We benchmarked the *IMCC*_*1*_ of different ρ values based on *JS*, and the *IMCC*_*1*_ scores were plotted vs. the observed *JS* for each module pair (Supplementary Table S3). Through nonlinear curve estimating and coefficient of determination (*R* ^*2*^) comparison, we quantitatively identified the best ρ value and the most fitting model. To obtain more precise results, we removed the outliers.

Our results suggested that the optimal ρ value was 1/1, and the most fitting model was logarithmic model with a *R* ^*2*^ of 0.616. Thus, the final formula for *IMCC* could be simplified as:

IMCC=SW+CT. (9)

We also plotted the *IMCC*_*2*_ against *JS* and compared *R* ^*2*^ of fitted curves of *IMCC*_*1*_ and *IMCC*_*2*_ to select the optimal integrative method.

### Comparison and Verification of IMCC

As many established algorithms addressed the problem of inter-module interaction evaluation using sum of weight (*SW*) of inter-module interactions, we compared SW with *IMCC* by fitting the KEGG database coverage. We also compared IMCC with inter-module average shortest path (*IMASP*), also named as inter-module average characteristic path length, a topological parameter proposed to evaluate the distance of a module pair. *IMCC* was plotted vs. the *IMASP* (Supplementary Table S4), and determination coefficient of curve fitting was also calculated.

## Global Transition of Module Rewiring and Local Pathological Module Pairs

### Global Transition of Module Rewiring

To access the transitions of inter-module coordination response to drug perturbation, we compared the distribution of IMCC score between the treated and untreated group by dividing these scores into four intervals by quartiles, through the chi-square test. Besides, the principal component analysis (PCA) was employed to evaluate the distance of IMCC distributions among these groups.

### Local Pathological Module Pairs Identification

In order to further investigate the core factors of the modular map, we tried to identify the “connectors.” We utilized three computational methods, that is, betweenness centrality, variation of ratio of characteristic path length and density (VRCD), and distribution of edge weight, to identify candidate connector modules from module networks. In the first method, the betweenness of each module in modular map was calculated, and the top 10% modules were selected as connectors. As for the second method, the VRCD was calculated to detect connectors. As the bridging modules constituted the channels between modules, their removal would lead to interruption of the inter-module connectivity (Missiuro et al., 2009; Yang et al., 2009; Zhu et al., 2014). Therefore, we removed nodes (module) in module map one by one and calculated the change ratio of characteristic path length and network density.

η=Δs/Δt, (10)

$$\Delta s=\frac{CPLa-CPLo}{CPLo},$$ (11)

$$\Delta t=\frac{Da-Do}{Do}.$$ (12)

Δs is the change ratio of characteristic path length of modular map. *CPL*_*o*_ is the origin characteristic path length. *CPL*_*a*_ is the altered characteristic path length of modular map after deleting a module. Δ*t* is the change ratio of density of modular map. *D*_*o*_ is the origin density. *D*_*a*_ is the altered density of modular map after deleting a module. η is the ratio of Δ*s* and Δ*t*.

We set the double average η in each modular map as respondent cutoff. Thereby, the modules, whose η is more than cutoff, were identified as connectors based on *VRCD*.

In the edge weight distribution, the inter-module connections were distributed based on their weight. The modules, whose inter-module connection weight is more than 0.1, were selected as connectors.

The connectors identified by all the three methods were regarded as the characteristic modules in corresponding state. A pair of connector modules (blue and brown) in the vehicle group was identified as characteristic pathological inter-module connection, which was named as “pathological module pair (PMP).”

### Calculation of Dissociation Rate of PMP

The dissociation rate (DR) of PMP was also calculated. We defined DR as the corrected ratio of the amount of modules before dissociation to that after dissociation. The formula of DR is as follows:

$$DR=\frac{n_{B}}{n_{A}}•\frac{N_{A}}{N_{B}}.$$ (13)

*n*_*A*_ is number of modules in the initial state (disease state, *n*_*A*_ = 2); *n*_*B*_ is number of modules in the succeeding state (treated by drugs); and *N*_*A*_ and *N*_*B*_ are the total number of modules in initial and succeeding states, respectively.

### *In Vivo* Experiments Validation

In order to verify the conclusion, we designed three validation assays using a rat MCAO model, which were introduced as the above section.

### Western Blot

Twenty MCAO rats were divided into five groups: sham, vehicle, BA, JA, and UA group and administrated as before. The hippocampus of rats was removed from their brains. After protein extraction and protein quantitative analysis, protein concentration was adjusted for load. We separated proteins using sodium dodecyl sulfate–polyacrylamide gel electrophoresis (SDS-PAGE). After proteins electrotransferred to nitrocellulose membranes, blots were incubated with rabbit anti-Map2k6 antibody (1:2,000, Santa Cruz, CA, United States) for overnight at 4°C. Then, cyclic membrane was washed and stained by goat anti-rabbit IgG. The membranes were incubated with an electrochemiluminescence reagent, After exposure, the band density was determined with a GS-700 densitometer (Bio-Rad). Each measurement was taken in three replicates.

### RT-PCR

All of 40 rats were grouped and administrated as mentioned in the earlier section. Total RNA from rats’ hippocampus was isolated using TRIzol (Invitrogen, United States). Real-time PCR was performed using a ABI 7300 Real-Time PCR System. The relative expression of BBC3 and Bcl2l1 was analyzed using the relative content, through the 2−^△△^Ct method normalized by GAPDH expression.

### Co-Immunoprecipitation (Co-IP)

Co-IP was performed to prove the interaction between BBC3 and Bcl2l1 as described in (Wang et al., 2009). Briefly, tissues from animals were homogenized. The procedure of supernatants collection and separation is the same as Western blot. After protein extraction and protein quantitative analysis, rabbit anti-BBC3 (CST14570, Danvers, MA, United States) and anti-Bcl2l1 (CST2764, Danvers, MA, United States) at a concentration of 1:1,000 and 1:2,000, respectively, were coupled to SiezeX beads according to the protocol of kit parameters (Pierce). Finally, eluted proteins were separated in 12–15% SDS-PAGE and electrotransferred to nitrocellulose for immunoblots.

## Results

### Drug Efficacy Variation in Ischemic Infarct Volume

According to the drug efficacy examination, BA, UA, and JA significantly reduced the ischemic infarct volume, whereas no significant change was detected in the CM group compared with vehicle. Therefore, we described BA, UA, and JA as effective drugs, and CM as an ineffective drug in the following section.

### Construction and Evaluation of Inter-module Coordination Analysis

**FIGURE 2:**
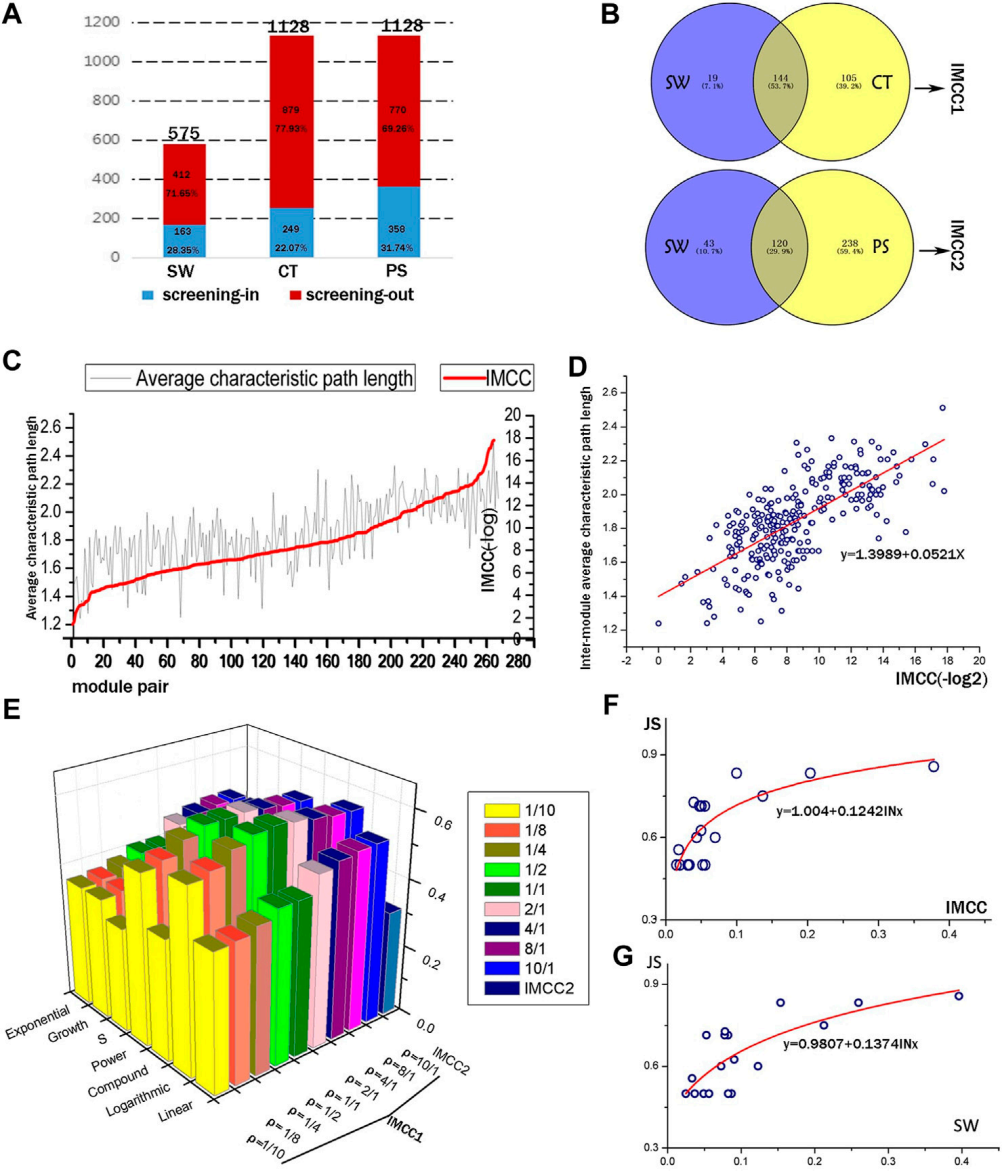
Quantitative evaluation of the IMCC method. **(A)** The screening outcomes of the three inter-module connectivity parameters (SW, CT, and PS). The height of the column represents the total amount of SW, CT, and PS, respectively. Red and blue parts of the columns represent the screened out and the remaining parameters, respectively. **(B)** The overlapping condition of two parameters to be integrated as IMCC_1_ and IMCC_2_. **(C)** The IMCC value against the inter-module average characteristic path length (IMASP) of module pairs. **(D)** Scatter plot and linear fitting of log2 transformation of IMCC vs. IMASP. The formula is y = 0.0521x + 1.3989, with *R* ^2^ = 0.493. **(E)** The *R* ^2^ of multiple fitting models for IMCC and JS. **(F,G)** are the fitting curves of logarithmic model of JS and IMCC, JS, and SW.

Based on the expression of above 374 genes, co-expression network was constructed and modules were identified using a WGCNA R package (Langfelder and Horvath, 2008). A total of 48, 23, 42, 15, and 24 modules were detected in the vehicle, BA, JA, UA, and CM groups, and the detailed process was described in our previous study (Li et al., 2016) (Supplementary Figure S1); the expression level of mRNA was shown in Supplementary Material 1. Inter-module coordination was achieved not only by direct interactions among modules but also through shared partners (Hsu et al., 2011) or between-module (or pathways) paths that consist of multiple proteins and interactions. In this study, we used the CT score and PS to evaluated inter-module relationship mediated by paths and SW for direct module-to-module interactions. For the false-positive levels inherent in the DNA microarray data, we introduced the hypergeometric distribution test, as a result, identified 28.35% SW and 31.74% PS with significance, and screened 22.07% valid CT score by cutoff value (Figure 2A; Supplementary Tables S1, S2). These CT scores and PS provided supplementary inter-module relationship: 64.42 and 146.01% more than SW (Figure 2B).

All the inter-module correlations summarized or calculated are presumed to contribute to biological function coordination. In this study, we employed KEGG signaling to optimize the IMCC (Supplementary Table S3). Our results (Figures 2E,F) suggested that the optimal ρ value in the IMCC_1_ model was 1/1 and the most fitting model was the logarithmic model with *R* ^2^ reaching the peak of 0.616 with two sides sloping down to lower values: when ρ = 1/10 or ρ = 10/1, the minimum *R* ^2^ of each side was observed, respectively (Supplementary Figure S2). Therefore, it is proper to decide that the integrated parameter IMCC_1_ is more consistent with the KEGG classification than any single index (SW or CT), which would provide more accurate evaluation of the relationship between modules. Using the same method, we compared the IMCC_2_ with IMCC_1_ and chose the IMCC_1_ model as the optimal model with a ρ value of 1/1. This also indicated that the quantitative parameter IMCC_1_ reflects the functional coordination of module pairs.

For comparison between SW and IMCC, the fitting model of SW was y = 0.1374ln(10) + 0.9807, with *R* ^2^ equal to 0.580, which is lower than 0.616 of IMCC (Figure 2G). The results indicate that IMCC achieved better performance on the weighted gene co-expression data. It also means that the results based on IMCC are more consistent with biological function than *SW*. This can be attributed to the adjustable parameters (weighted coefficient α and β) for SW and consistency score (CT), which make IMCC more likely to reach the optimal result in KEGG function coverage.

From the topological structure, the *IMCC* was generally consistent with inter-module average shortest path with coefficient of determination equal to 0.493 (Figures 2C,D; Supplementary Table S4).

## Global Modular Map Rewiring Reveals the Perturbation Pattern of Drugs on Disease

### Drugs Destroyed the Characteristic “Core–periphery” Structure in Disease

**FIGURE 3:**
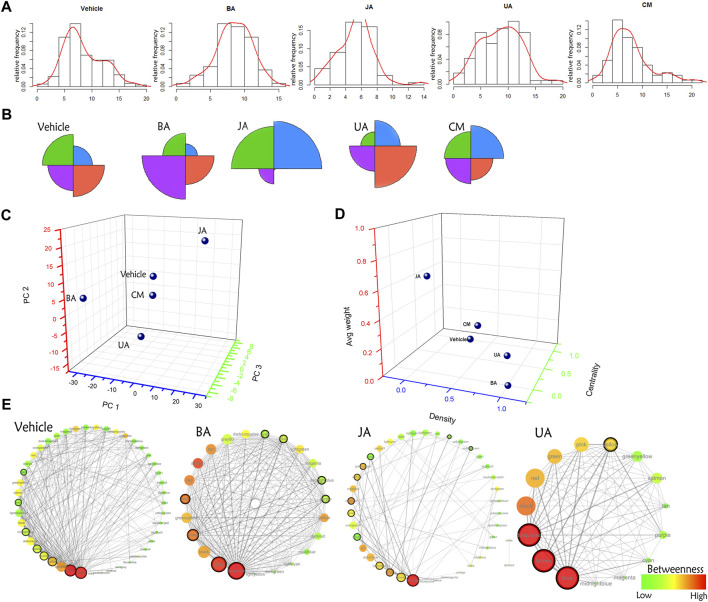
Comparison of distribution of the IMCC score and global modular map rewiring. **(A)** The relative distribution of the IMCC score. The *X*-axis is the -log2 of the IMCC score. **(B)** Star graph of the relative account of the IMCC score distributing in four intervals (−∞, 5.2] (5.2–7.3] (7.3–10.2] (10.2−+∞]. These four intervals were indicated by black, red, green, and blue, respectively. **(C)** The principal component analysis of distribution of the IMCC score in each group. Each interval is set as variable, and relative frequencies in each interval are set as values of corresponding variables. **(D)** Euclidean distance of architecture of modular map in different groups. Three indexes (average weight, density, and centrality) were used to constitute the dimensions of space euclidean distance calculation. **(E)** Modular map based on IMCC across conditions. Each circle in modular map represents a module, the color of which indicates the betweenness in the corresponding modular map. Circles with black solid line around are connectors (modules) identified by edge weight distribution for further GO enrichment analysis.

Globally, the distribution of IMCC between disease and treated groups was obviously diverse (Figures 3A,B): BA-, JA-, and UA-treated groups were different compared to the vehicle group with significance (chi-square test, *p* < 0.05), whereas no statistical difference was noted between the ineffective (CM) group and the vehicle group (chi-square test, *p* = 0.06374). Besides, according to the distance in PCA, CM was the nearest to the vehicle (Figure 3C; Supplementary Table S5). This indicated that the transitions of inter-module coordination induced by the effective drugs were more thorough than those induced by the ineffective one.

Based on the IMCC, the modular map across condition was constructed in which modules were regarded as uniform nodes and the IMCC between any pair of modules as edges. As a result, the module map provided a holistic perspective of the inter-module coordination and framework extracted as “backbone” in disease and treated conditions. According to the topological analysis, the modular map in the disease exhibited a topical “core–periphery” structure, with the network centrality of the modular map of 0.525. Whereas this “core–periphery” structure collapsed in the treated groups, the network centrality was significantly decreased to 0.156, 0.271, and 0.22 (one-sample *t*-test, two-sided *p* < 0.05) in BA, JA, and UA groups, respectively (Figures 3D,E; Table 1).

**TABLE 1:** Topological parameters of the module map in different groups. The symbols “↑” and “↓” represent “increase” and “decrease,” respectively, compared with the vehicle group. The three highlighted parameters with red letter were selected as the representative index of the three types of parameters for further euclidean distance calculation.

Group	Avg. degree	Density	Characteristic path length	Diameter	Avg. betweenness	Cluster coefficient	Centrality	Avg. weight	Euclidean distance
Vehicle	11.911	0.271	1.848	4	0.0197	0.615	0.525	0.0213	–
BA	18.87↑	0.858↑	1.142↓	2↓	0.0068↓	0.867↑	0.156↓	0.0208↓	1.2655
JA	3.95↓	0.101↓	2.865↑	7↑	0.0493↑	0.281↓	0.271↓	0.081↑	0.9476
UA	11.333↓	0.81↑	1.19↓	2↓	0.0147↓	0.857↑	0.22↓	0.0357↑	1.0998
CM	8.571↓	0.429↑	1.71↓	4	0.0373↑	0.661↑	0.411↓	0.0409↑	0.4199

It is indicated that from the mesoscopic perspective, network organization in stress exhibits a characteristic “core–periphery” architecture. In this architecture, the “periphery” is constituted of small well-defined communities; conversely, the “core” consists of highly interconnected larger modules harder to decompose (Bollobás, 1998; Lancichinetti et al., 2010; Han et al., 2013; Verma et al., 2016). According to results, in this “core–periphery” structure in disease condition, all the information propagation is of radial distribution around a couple of modules, and the integrity of the network is completely dependent on the modules at the center; thus, the system is liable to collapse under stress response. It is also suggested that when a biological network is on stress, the center of this “core–periphery” structure is a conserved and stable module, with crucial role for cell survival rather than development (Mihalik and Csermely, 2011; Kim et al., 2012; Han et al., 2013), to adapt to the novel situation. Accordingly, the modular map in disease state exhibits a characteristic “core–periphery” structure, core of which may contribute to cell survival. And this structure would be rewired and collapse in response to drug perturbation.

### Aggregation and Dispersion of Modules: Two Allosteric Directions of Drugs

Although the network centrality decreased in all of the treated groups, we wondered if there were any distinct topological structures in different effective drug groups. Using analysis of neighborhood-based parameters in modular maps, we noted that the effective drugs showed two distinct regulatory directions. For one direction, in BA and UA groups, the inter-module connections were very dense and modules tended to aggregate. For example, the network density increased from 0.271 in vehicle to 0.858 (increased by 216.6%) and 0.81 (increased by 198.9%), the characteristic path length of the modular map decreased from 1.848 in vehicle to 1.142 (decreased by 38.2%) and 1.19 (decreased by 35.6%), in BA and UA groups, respectively. Generally, after treatment with BA and UA, the centrality of the modular map decreased, and the connections between noncentral modules increased (Figures 3D,E and Table 1). All these alterations drive the modular map into a clique-like structure, in which any module pairs are connected by edges. Such alterations indicated that information propagation was easier to be implemented. Such a structure is shown to have the highest integrity and the strongest robustness under perturbation. Overall, the modules in BA or UA groups aggregated into a more intensively connected integrity.

As for the opposite direction, in the JA group, the modular map altered into a sparser network, and the information propagation path was elongated: compared with vehicle, the network density decreased to 0.101 (decreased by 62.7%); and the characteristic path length and average betweenness centrality increased by 55 and 150%, to 2.865, and 0.0493, respectively. In general, after treatment with JA, the centrality of the modular map also decreased, which attributed to the disruption between central modules and noncentral modules. Thus, we assumed that the effect of JA on the module map included module dispersing. It should be noted that the average weight nearly quadrupled from 0.021 in vehicle to 0.081 in JA (Figures 3D,E and Table 1), indicating that the paths between modules became wider and smoother than those in disease state. Therefore, we inferred that the pharmacological effects of JA were decoupling partial inter-module paths and widening the remaining connectivity, which may consequently interrupt the pathological information transmission and enhance the remaining information transmission.

Both of module aggregation and dispersion effect of drugs destroyed the characteristic “core–periphery” architecture in disease, that means the importance of “core” for cell survival decreased in treated conditions, and the emphasis of the cell may turn from survival into development. We can infer that cerebral ischemia injury was repaired after treated by these drugs. Then, what is the biological function of the “core”? How is this “core” reversed by these drugs?

## Local Dissection of Cooperative Pathological Module Pair Indicated Mechanisms

### Identification and Dissection of Connectors

**FIGURE 4:**
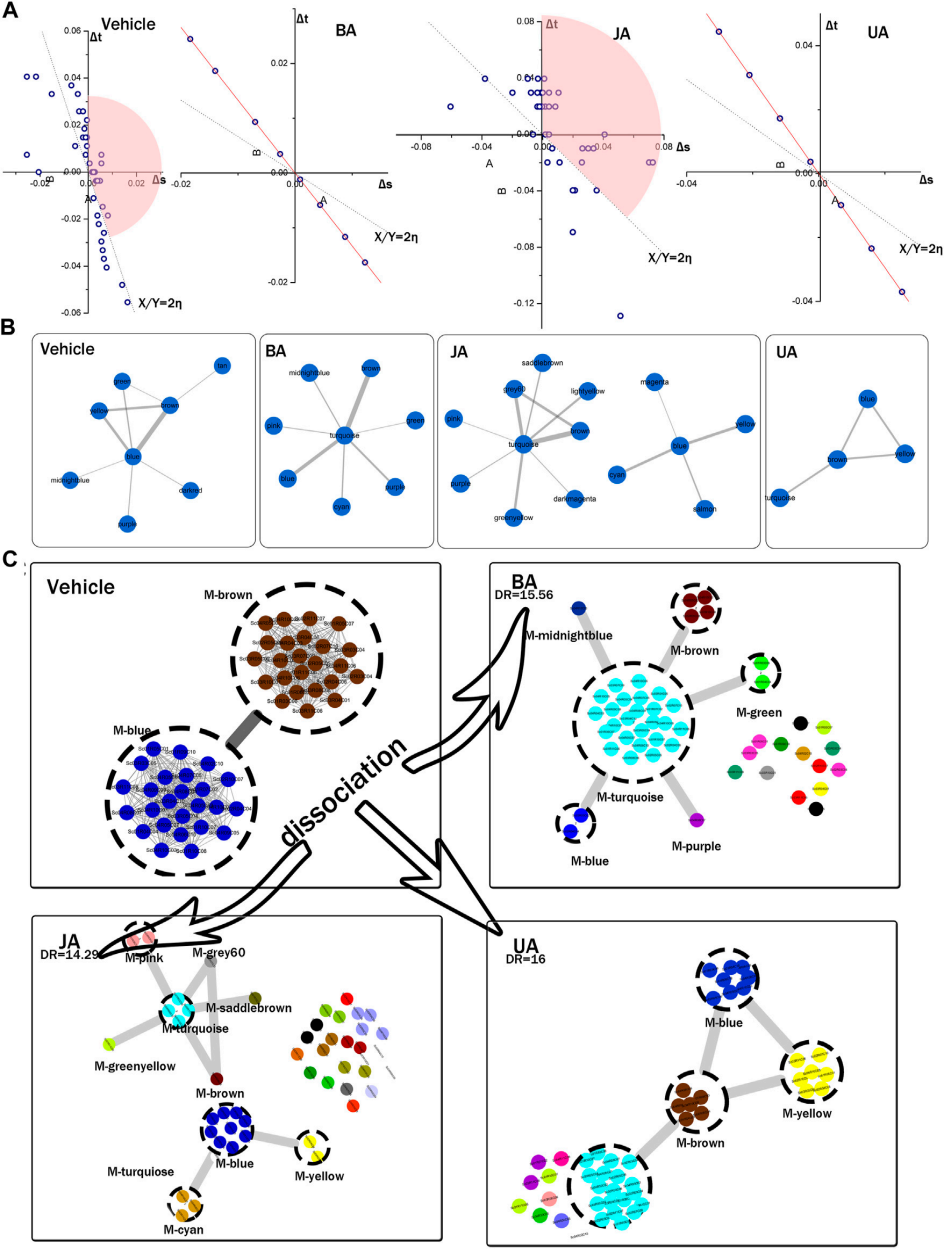
Identification of connectors and dissociation of pathological module pair. **(A)** Connectors identified by variation of ratio of characteristic path length and density (VRCD). The Δs and Δt represent the variation ratio of characteristic path length to network density, η represents average ratio of Δs and Δt. The 2-fold change of η, shown as dotted linear, was set as cutoff. Modules in the pink sector domains were identified as connectors. None of modules were identified as connector in BA and UA groups for their clique-like structure with equal η of all modules (red line). **(B)** Connector sub-network identified by edge weight distribution. **(C)** Local dissection of cooperative pathological module pair (PMP). Genes of PMP in vehicle were scattered into different modules in BA-, JA-, and UA-treated groups. Each dashed circle represents a module; circles with the same color in the same region indicate genes in the same module; gray rough lines represent the inter-module connections; gray thread lines represent the interactions between genes.

The topological structure analysis of modular map provides a landscape of the transition of inter-module connections from disease to treated conditions. In order to identify the modules contributing to information propagation in the modular map, we employed three methods to detect the “connectors” (Figure 4A, Supplementary Tables S6, S7), which were defined as modules bridging one community to another (Yu et al., 2007; Zhu et al., 2014) and controlling the information flow dissemination between modules. As a result, connector networks were detected in each group (Figure 4B). In the light of the disease condition, the module pair with the strongest coordination was identified as connectors, which can be regarded as “a broad bridge with heavy traffic, controlling essential pass.” This pair of connectors can be treated as the “core” reflecting the characteristic pathological process in the disease condition (Figure 4C), so were named as “pathological module pair (PMP).”

To assess the allosteric communication of connectors from untreated to treated conditions, we investigated what had happened to the “core” after perturbation to reveal the pharmacological mechanisms of these drugs. Dissociation rate (DR) was proposed to evaluate how one modular pair in one condition scattered in other condition. The PMP in the vehicle group dispersed into several modules in different treated groups. The DR was 15.65, 14.29, and 16 in BA, JA, and UA, respectively (Table 2). As shown in Figure 4C, the effective drugs also displayed two distinct directions. In the BA group, more than one half of the nodes constituting PMP dispersed into one common module (turquoise). Similar to BA, 81.63% nodes in the PMP dispersed into four connectors (blue, brown, turquoise, and yellow) after treatment with UA. Nodes from the PMP aggregated into a compact integrity, like an intensely interacted module sub-network facilitating information communication. In the JA group, the nodes from the PMP scattered into 25 modules, the amount of which accounted for more than one half of the total 48 modules. This dispersion from an intimate interaction to more sparsely association was also noted in the modular map analysis, which would lead to interruption of some information propagation.

**TABLE 2:** Dissociation rate of the PMP in each treated group.

	BA	JA	UA	CM
nA	2	2	2	2
nB	15	25	10	11
NA	48	48	48	48
NB	23	42	15	23
DR	15.65	14.29	16	11.48

### PMP Dissociation Leads to Reversion of the Pathological Processes

In order to understand how functions altered by PMP dispersion, we enriched KEGG pathways for each module of the PMP and modules that PMP nodes assembling in treated condition (Table 3).

**TABLE 3:** Enriched KEGG pathways of the PMP in disease condition, and top 10 KEGG pathways of modules in BA and UA that PMP nodes scattered in.

	Enriched KEGG pathways	*p* value
Vehicle-blue	mmu04150: mTOR signaling pathway	0.010,800,621
	mmu04720: Long-term potentiation	0.017,728,358
	mmu04270: Vascular smooth muscle contraction	0.048,072,485
Vehicle-brown	mmu05014: Amyotrophic lateral sclerosis	3.13E-04
	mmu04010: MAPK signaling pathway	0.00308,276
	mmu05020: Prion diseases	0.00314,163
Top 10 signaling of BA-blue	mmu05200: Pathways in cancer	3.40E-16
	mmu04912: GnRH signaling pathway	4.48E-15
	mmu04010: MAPK signaling pathway	9.55E-14
	mmu05166: HTLV-I infection	4.56E-11
	mmu05161: Hepatitis B	2.49E-10
	mmu04722: Neurotrophin signaling pathway	1.95E-09
	mmu04062: Chemokine signaling pathway	2.59E-09
	mmu04668: TNF signaling pathway	4.82E-08
	mmu05203: Viral carcinogenesis	1.91E-07
	mmu05210: Colorectal cancer	3.01E-07
Top 10 signaling of UA-blue, brown, turquoise, and yellow	mmu05200: Pathways in cancer	3.40E-16
	mmu04912: GnRH signaling pathway	4.48E-15
	mmu04010: MAPK signaling pathway	9.55E-14
	mmu05166: HTLV-I infection	4.56E-11
	mmu05161: Hepatitis B	2.49E-10
	mmu05212: Pancreatic cancer	1.54E-10
	mmu04722: Neurotrophin signaling pathway	1.95E-09
	mmu04062: Chemokine signaling pathway	2.59E-09
	mmu04015: Rap1 signaling pathway	1.53E-09
	mmu04668: TNF signaling pathway	4.82E-08

All enriched KEGG pathways of the PMP in disease are related to the pathological process of the nervous system injury. For example, inhibition of MAPK signaling or mTOR signaling can effectively reduce cerebral ischemia reperfusion injury (Dennis et al., 2001; Sovan, 2013; Johansson et al., 2014; King et al., 2015; Yang et al., 2016). Ischemic long-term potentiation (i-LTP) increasing and vascular smooth muscle contraction are pathological changes following cerebral ischemic injury (Maddahi et al., 2009) (Orfila et al., 2014) (Wang et al., 2014). ALS is related to motor neurons degeneration in the cortex (Sathasivam et al., 2001); (Menzies et al., 2002); (Hristelina et al., 2009); (Jordi 2009 Giovanni 2009); (Ivanova et al., 2014). Prion disease may lead to neuronal death by oxidative stress and regulation of complement activation (McLennan et al., 2004). Notably, these pathways were all cross-talk with MAPK signaling: MAPK signaling pathway is the upstream signaling of mTOR signaling pathway and is involved in long-term potentiation and ALS and also acts as a critical factor to mediate vascular smooth muscle contraction. It is the inter-module structural connection in PMP that bridges the functional correlations among these nervous injury–related pathways, in which the MAPK signaling pathway plays a major role. Therefore, these pathways of PMP reflected the cell survival condition in the process of neural ischemia injury.

Whereas, in the treated groups, PMP was dissociated into several modules, accompanied with reversion of the pathological processes and a new adaptive balance of biological system. In BA and UA, the top 10 KEGG signaling concentrated in MAPK signaling, GnRH signaling, viral infection, cancer, neurotrophin signaling, and chemokine signaling pathway. That means, after treated, the emphasis of the cell shift from survival, represented by nervous injury–related pathways centered on MAPK signaling, into development and repair, such as neurotrophin regulation, chemokine signaling, and hormone releasing.

### Assay Validation

To validate the PMP that is essential for the contribution of disease phenotype, we used MCAO rats to examine the core protein, genes, and interaction. First, we compared the expression of a protein (MAP2k6) of MAPK signaling by Western blot in the disease and treated groups. Second, in order to verify the regulatory direction of BA, JA, and UA, we used RT-PCR to examine the gene expression differences of a pair of genes from PMP, Bcl2l1, and BBC3. The interaction of the two genes was tested using immune co-immunoprecipitation (co-IP).

**FIGURE 5:**
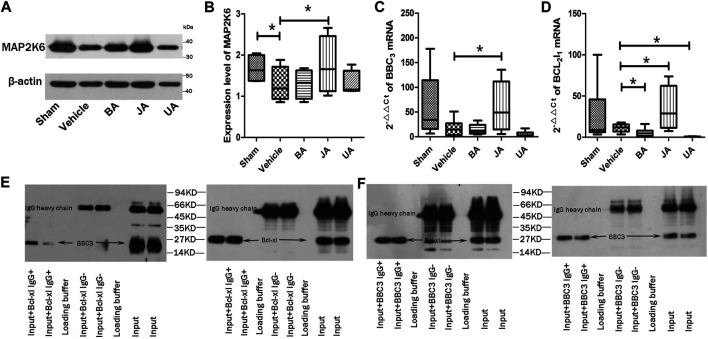
Validation of core protein, genes, and interaction in PMP. **(A and B)** show expression of MAP2K6 of Western blot in distinct groups. **(C and D)** are relative contents of BBC3 and Bcl2l1 among different groups. *p < *0.05*. **(E and F)** represent the co-immunoprecipitation results of BBC3 and Bcl-xl.

According to Western blot analysis (Figures 5A,B, Supplementary Figure S3), the expression of Map2k6 protein in mice brains was significantly decreased in the vehicle group than the sham group (*p* < 0.05, one-sided, paired *t*-test). Expression of Map2k6 protein increased significantly in the JA group compared with samples in the vehicle group (*p* < 0.05, one-sided, paired *t*-test). No statistically significant differences were found in other treated groups.

The BBC3 and Bcl2l1 mRNA were examined by RT-PCR (Figures 5C,D). The BBC3 mRNA expression was significantly increased in the JA group (*p* < 0.05, one-sided, paired *t*-test), but decreased slightly in BA and UA groups. Compared with vehicle, the expression of Bcl2l1 was significantly increased in the JA group (*p* < 0.05, one-sided, paired *t*-test) but decreased significantly in BA and UA groups (*p* < 0.05, one-sided, paired *t*-test). The outcome of RT-PCR indicated that the regulatory effects of BA and UA showed opposite directions to that of JA.

Both Bcl-Xl and BBC3 were detected in precipitation products of BBC3 IgG or Bcl-Xl IgG (Figures 5E,F). There was an interaction between BBC3 protein and Bcl-XL protein. By searching the String database (String 10.0, http://www.string-db.org/), we found that BBC3 and Bcl2l1 were correlated with each other (correlation score = 0.999), remarked by reaction and binding.

## Discussion

In the current study, we proposed an integrated mathematical model for quantitative evaluation of the transition of inter-module coordination across conditions. Globally, we found the high centrality “core–periphery” modular map is a characteristic structure in cerebral ischemia condition. Furthermore, it is the rewiring of the interactions between modules and consequent architecture alteration in the modular map, rather than a single gene or a single module, that leads to phenotype alteration in response to drugs perturbation. Our result also indicated that BA, UA, and JA showed diversification of the pharmacological effect directions: the pharmacological mechanism of BA and UA can be attributed to aggregation of modules into a clique-like community, enhanced connections between noncentral modules, facilitation of paths for information communication between non-modules, and promotion of robustness in response to perturbation; the pharmacological mechanism of JA is ascribed to selective interruption of information transmission and enhancement of some specific information propagation. According to the local allosteric, the “core” of “core–periphery” structure is an interacting PMP, whose biological function focused on nervous injury pathways bridged by MAPK signaling pathway. BA and UA aggregated the dissociated nodes into a compact integrity, whereas JA dispersed the dissociated nodes from the PMP. PMP dissociation leading by drugs contributed to the reversion of the pathological condition: the focus of the cellular function shift from survival after nervous system injury into development and repair, including neurotrophin regulation, hormone releasing, and chemokine signaling activation. Finally, these results were validated by *in vivo* experiments of MCAO rats.

Ischemic stroke is a complex disease with multiple gene mutations. For this disease, a "from systematic to molecular levels" analytical strategy was established to decipher the pharmacological mechanism of multi-target drugs. Our result highlights the holistic inter-module coordination rearrangement rather than a target or a single module that brings phenotype alteration. That may indicate in the biological system, one kind of complex adaptive system composed of a set of basic units and interactions, rewiring of interactions between basic units lead to nonlinear phenomena: novel phenotype emergence to response to perturbation (Zhang et al., 2014). Therefore, complexity, in essence, is a science of emergence. The challenge is how to discover the primary principles of emergence, as a foundation, to quantify the interactions between basic units and their alterations across different conditions. Quantitative analysis of drug-induced transition in modular map was the principal problem for systematically elucidating the comprehensive pharmacological mechanisms of multi-target drugs and the inevitable choice for network-based drug discovery. Our strategy may help to map the detailed variations of inter-module pharmacological actions of multiple-target drugs and could be set as reference for other active ingredients.

## Data Availability

The datasets presented in this study can be found in online repositories. The names of the repository/repositories and accession number(s) can be found in the article/Supplementary Material.

## Abbreviation

AKT, protein kinase B; ALS, amyotrophic lateral sclerosis; BA, baicalin; cAMP, cyclic adenosine monophosphate; CM, concha margaritifera; CT, consistency score; DIMC, direct inter-module connections; DR, dissociation rate; ECA, external carotid artery; GnRH, gonadotropin-releasing hormone; IIMC, indirect inter-module connections; IMASP, inter-module average shortest path, also named as inter-module average characteristic path length; IMCC, inter-module coordination coefficient; JA, jasminoidin; JS, Jaccard similarity; KEGG, Kyoto Encyclopedia of Genes and Genomes; LTP, long-term potentiation; MAPK, mitogen-activated protein kinase; MCAO, middle cerebral artery obstruction; mTOR, mammalian target of rapamycin; PCA, principal component analysis; PI3K: phosphoinositide 3-kinases; PMP, pathological module pair; PS, path strength; Rap1, Ras-proximate-1; SW, sum of inter-module weight; TTC, 4% 2,3,5-triphenyltetrazolium chloride; UA, ursodeoxycholic acid; VRCD, variation of ratio of characteristic path length and density
