# Supplementary material for: IMCC: A Novel Quantitative Approach Revealing Variation of Global Modular Map and Local Inter-Module Coordination Among Differential Drug’s Targeted Cerebral Ischemic Networks
Source: Front Pharmacol. 2021 Apr 16;12:637253. doi: 10.3389/fphar.2021.637253 (PMC8087074; doi:10.3389/fphar.2021.637253)
Supplement: Supplementary file 1 [file datasheet1.zip › Supplementary table 1-7/supplementary table 1-7.docx]

Supplement table 1

| **M_x_** | **M_y_** | **CT'** | **CT** | **SW'** | **SW** | **P-value** |
| --- | --- | --- | --- | --- | --- | --- |
| bisque4 | blue |  |  | 0 | 0.027148007 | 0.027745373 |
| bisque4 | greenyellow | 0.000110933 | 13.0552926 |  |  |  |
| bisque4 | lightgreen |  |  | 0.005820082 | 0.228652375 | 0.00665549 |
| bisque4 | red | 0.000377807 | 20.11151106 | 0.010854517 | 0.402955909 | 0.003585366 |
| bisque4 | yellowgreen |  |  | 0.003893454 | 0.161948159 | 0.038866039 |
| black | blue | 0.028267952 | 757.5354614 | 0.082096444 | 2.869512267 | 4.11582E-09 |
| black | brown | 0.008431805 | 233.0616821 |  |  |  |
| black | cyan | 0.000559784 | 24.92305825 |  |  |  |
| black | darkgreen | 0.000296399 | 17.95906577 |  |  |  |
| black | darkorange | 1.47991E-05 | 10.51348415 |  |  |  |
| black | darkred | 0.000498899 | 23.31323147 |  |  |  |
| black | darkturquoise | 0.001065835 | 38.30319727 |  |  |  |
| black | greenyellow | 0.00097363 | 35.86527705 |  |  |  |
| black | lightcyan1 | 0.000709852 | 28.89088311 | 0.008033836 | 0.305297553 | 0.006022613 |
| black | lightgreen | 0.013504429 | 367.1834148 | 0.079294323 | 2.772496505 | 7.202E-27 |
| black | lightsteelblue1 | 5.85543E-05 | 11.67038548 | 0.01424866 | 0.520468804 | 0.000540977 |
| black | magenta | 0.001944564 | 61.53704622 |  |  |  |
| black | orange | 0.00212843 | 66.39853575 | 0.016528362 | 0.599397238 | 0.002424233 |
| black | orangered4 | 0.002672461 | 80.78287386 | 0.02188874 | 0.784985612 | 1.30859E-08 |
| black | pink | 0.003808215 | 110.8125618 | 0.051247697 | 1.801458994 | 3.07547E-05 |
| black | purple | 0.004457484 | 127.9794407 | 0.032605904 | 1.156038035 | 6.16949E-05 |
| black | royalblue | 6.99423E-06 | 10.30712057 |  |  |  |
| black | turquoise | 0.046417308 | 1237.409967 | 0.153617144 | 5.345720402 | 2.75117E-15 |
| blue | brown | 1 | 26450.42719 | 1 | 34.64940606 | 2.7807E-303 |
| blue | brown4 | 0.005397073 | 152.8224582 | 0.051916851 | 1.824626604 | 3.1505E-15 |
| blue | cyan | 0.001933134 | 61.23485132 |  |  |  |
| blue | darkolivegreen | 0.018150727 | 490.0329582 | 0.07307461 | 2.55715602 | 4.84438E-21 |
| blue | darkorange | 0.013880513 | 377.1271886 | 0.072688453 | 2.543786375 | 2.05078E-13 |
| blue | darkorange2 | 0.000241554 | 16.50894285 |  |  |  |
| blue | darkred | 0.059297136 | 1577.956561 | 0.249270797 | 8.657465877 | 7.99678E-76 |
| blue | darkslateblue | 0.005879088 | 165.5670693 | 0.056852073 | 1.99549516 | 7.8678E-22 |
| blue | green | 0.148299497 | 3931.206122 | 0.259532404 | 9.012745855 | 3.41057E-72 |
| blue | grey60 | 0.008896189 | 245.3401299 | 0.090608714 | 3.164226283 | 1.19042E-11 |
| blue | lightcyan1 | 0.000366375 | 19.80925447 |  |  |  |
| blue | lightgreen | 0.0236589 | 635.6707349 | 0.079504221 | 2.779763654 | 6.71638E-14 |
| blue | lightsteelblue1 | 0.01122292 | 306.8596312 | 0.064153355 | 2.248282023 | 8.6802E-31 |
| blue | lightyellow | 0.004812127 | 137.3562869 | 0.037614422 | 1.329444245 | 0.002398754 |
| blue | magenta | 0.01199521 | 327.279208 | 0.082106527 | 2.869861358 | 4.05401E-09 |
| blue | midnightblue | 0.060671155 | 1614.286031 | 0.212475298 | 7.383522589 | 2.64843E-66 |
| blue | orange | 0.003600898 | 105.3310248 |  |  |  |
| blue | orangered4 | 0.001084279 | 38.79086719 |  |  |  |
| blue | paleturquoise | 0.000564205 | 25.03993024 | 0.022095106 | 0.792130483 | 0.043059035 |
| blue | pink | 0.008289852 | 229.3083972 | 0.087205421 | 3.046396611 | 1.61029E-09 |
| blue | plum1 | 0.002209341 | 68.53784014 | 0.021611253 | 0.775378401 | 0.001693134 |
| blue | purple | 0.078908544 | 2096.488149 | 0.276235286 | 9.591037369 | 5.30624E-80 |
| blue | royalblue | 0.000604717 | 26.11108798 |  |  |  |
| blue | salmon | 0.024224976 | 650.6379483 | 0.0868909 | 3.035507172 | 1.13338E-24 |
| blue | sienna3 | 0.001758866 | 56.62713278 |  |  |  |
| blue | skyblue |  |  | 0.032753883 | 1.161161389 | 0.005625076 |
| blue | skyblue3 | 0.0015049 | 49.91221364 | 0.019473565 | 0.701366816 | 0.016724637 |
| blue | steelblue | 0.000998188 | 36.51459772 |  |  |  |
| blue | tan | 0.031296014 | 837.5983588 | 0.056379743 | 1.979142002 | 2.58579E-09 |
| blue | yellow | 0.36006143 | 9530.25621 | 0.395964152 | 13.73632105 | 3.5238E-114 |
| blue | yellowgreen | 0.000173623 | 14.71282586 |  |  |  |
| brown | brown4 | 0.005115014 | 145.3647293 | 0.06379119 | 2.235743049 | 7.44294E-17 |
| brown | cyan | 0.005799843 | 163.4718204 | 0.056292265 | 1.976113322 | 4.67789E-09 |
| brown | darkgrey | 0.000397514 | 20.63259292 | 0.037147887 | 1.313291749 | 1.72502E-08 |
| brown | darkolivegreen | 0.011310841 | 309.1842752 | 0.023371643 | 0.836327062 | 0.015872627 |
| brown | darkorange | 0.016015881 | 433.5869749 | 0.122813101 | 4.279214885 | 5.69485E-37 |
| brown | darkorange2 | 0.000111664 | 13.07461688 |  |  |  |
| brown | darkred | 0.052821244 | 1406.731982 | 0.165751364 | 5.765834518 | 1.73871E-65 |
| brown | darkslateblue | 0.003602729 | 105.3794557 |  |  |  |
| brown | darkturquoise | 0.000837781 | 32.27336341 |  |  |  |
| brown | green | 0.096603458 | 2564.347075 | 0.134861177 | 4.696346461 | 6.16585E-20 |
| brown | greenyellow | 0.000538964 | 24.37255055 |  |  |  |
| brown | grey60 | 0.006399771 | 179.3340887 | 0.054244782 | 1.905224857 | 4.11253E-06 |
| brown | lightcyan1 | 0.000185548 | 15.02813002 |  |  |  |
| brown | lightgreen | 0.021213049 | 571.0016745 | 0.058418829 | 2.049739781 | 2.60827E-11 |
| brown | lightsteelblue1 | 0.009564366 | 263.0069451 | 0.046282038 | 1.629536668 | 9.27617E-22 |
| brown | lightyellow | 0.007326767 | 203.8441399 | 0.075717088 | 2.648644578 | 2.60827E-11 |
| brown | magenta | 0.008439488 | 233.2648243 | 0.034336338 | 1.215949549 | 0.019696443 |
| brown | midnightblue | 0.042665281 | 1138.20523 | 0.055526373 | 1.94959642 | 7.40254E-12 |
| brown | orange | 0.003902556 | 113.3069708 | 0.04300887 | 1.516212215 | 1.64021E-07 |
| brown | orangered4 | 0.000512054 | 23.66106783 |  |  |  |
| brown | pink | 0.000934641 | 34.83439149 |  |  |  |
| brown | plum1 | 0.001375584 | 46.49306145 |  |  |  |
| brown | purple | 0.061928444 | 1647.529129 | 0.158652476 | 5.52005498 | 3.62107E-42 |
| brown | red | 0.017291913 | 467.3256414 | 0.120030643 | 4.182879917 | 9.68811E-13 |
| brown | royalblue | 0.000245382 | 16.61017265 |  |  |  |
| brown | salmon | 0.021424902 | 576.6031381 | 0.0824748 | 2.882611803 | 4.47799E-21 |
| brown | sienna3 | 0.002066804 | 64.76911738 | 0.031900211 | 1.131605354 | 9.92252E-09 |
| brown | skyblue3 | 0.001438691 | 48.16160896 | 0.024409463 | 0.872258721 | 0.000383027 |
| brown | tan | 0.042455641 | 1132.662284 | 0.262464948 | 9.114277158 | 9.04103E-69 |
| brown | white | 0.000936754 | 34.8902442 | 0.024880265 | 0.88855897 | 0.035135175 |
| brown | yellow | 0.314452969 | 8324.3546 | 0.318186134 | 11.04347046 | 1.09561E-92 |
| brown | yellowgreen | 0.000165431 | 14.49624336 |  |  |  |
| brown4 | darkorange | 0.000102366 | 12.82878364 |  |  |  |
| brown4 | darkred | 0.000782757 | 30.81851929 | 0.00612618 | 0.239250197 | 0.002473631 |
| brown4 | green | 0.000765136 | 30.35263219 |  |  |  |
| brown4 | lightsteelblue1 | 3.43965E-05 | 11.03164569 |  |  |  |
| brown4 | midnightblue | 0.00069521 | 28.50375101 | 0.005262555 | 0.209349528 | 0.025717794 |
| brown4 | purple | 0.000823743 | 31.90220974 |  |  |  |
| brown4 | salmon | 0.000224471 | 16.05727678 |  |  |  |
| brown4 | tan | 0.000453926 | 22.124121 | 0.008492857 | 0.321189904 | 0.047560215 |
| brown4 | yellow | 0.0030119 | 89.75773342 | 0.037111366 | 1.312027291 | 2.15568E-09 |
| cyan | darkgreen | 0.00013156 | 13.60068331 | 0.01137946 | 0.421130595 | 0.040732034 |
| cyan | darkorange | 0.000869688 | 33.11700563 | 0.025642155 | 0.914937315 | 8.61209E-08 |
| cyan | darkred | 1.0812E-05 | 10.40806308 |  |  |  |
| cyan | darkturquoise | 0.000509873 | 23.60339844 | 0.017136903 | 0.620466299 | 0.002132851 |
| cyan | greenyellow | 0.001171855 | 41.10638298 |  |  |  |
| cyan | lightcyan1 | 0.000253335 | 16.82044823 | 0.015469803 | 0.562747518 | 3.28936E-06 |
| cyan | lightgreen | 0.00275352 | 82.92610144 | 0.023037797 | 0.824768575 | 3.52004E-07 |
| cyan | orange | 6.54633E-06 | 10.29527804 |  |  |  |
| cyan | white | 0.000223502 | 16.03165321 | 0.013935822 | 0.509637618 | 0.000656425 |
| darkgreen | greenyellow | 0.001051968 | 37.93654554 | 0.013331704 | 0.488721688 | 0.016373892 |
| darkgreen | lightcyan1 | 0.000140579 | 13.83914736 | 0.011120773 | 0.412174283 | 0.000419454 |
| darkgreen | lightgreen | 0.000887594 | 33.59043789 | 0.019719413 | 0.709878603 | 6.79333E-06 |
| darkgreen | orangered4 | 0.000329171 | 18.82557953 | 0.016207112 | 0.588274821 | 2.42733E-05 |
| darkgreen | red | 0.001420601 | 47.6833266 | 0.021418619 | 0.768708966 | 0.000971031 |
| darkgreen | skyblue3 |  |  | 0.008347826 | 0.316168606 | 0.003774427 |
| darkgreen | violet | 0.000249167 | 16.71024795 | 0.013522542 | 0.495328945 | 0.003774427 |
| darkgrey | greenyellow | 5.21789E-05 | 11.5018161 |  |  |  |
| darkolivegreen | darkred | 0.001947967 | 61.62703138 |  |  |  |
| darkolivegreen | darkslateblue | 0.000155875 | 14.2435647 |  |  |  |
| darkolivegreen | green | 0.009987302 | 274.1894903 | 0.072638233 | 2.54204767 | 2.52138E-23 |
| darkolivegreen | grey60 | 0.00073317 | 29.50744057 | 0.008820134 | 0.332520947 | 0.004445994 |
| darkolivegreen | lightgreen | 0.000146096 | 13.98500914 |  |  |  |
| darkolivegreen | lightsteelblue1 | 0.000662377 | 27.63564341 | 0.009311856 | 0.349545504 | 8.4515E-05 |
| darkolivegreen | midnightblue | 0.002863412 | 85.83167925 | 0.013405787 | 0.491286616 | 0.014083305 |
| darkolivegreen | purple | 0.00364594 | 106.5219437 | 0.030046636 | 1.067430408 | 7.85146E-07 |
| darkolivegreen | tan | 5.87716E-05 | 11.6761297 |  |  |  |
| darkolivegreen | yellow | 0.00449907 | 129.0789782 |  |  |  |
| darkorange | darkred | 0.002682392 | 81.04545261 | 0.015498748 | 0.563749672 | 1.92981E-05 |
| darkorange | green | 0.000850794 | 32.61745517 |  |  |  |
| darkorange | greenyellow | 4.52231E-05 | 11.31790452 |  |  |  |
| darkorange | lightgreen | 0.001599967 | 52.42581021 | 0.021951363 | 0.787153752 | 5.21736E-09 |
| darkorange | lightsteelblue1 | 0.000469685 | 22.54079963 |  |  |  |
| darkorange | magenta | 0.000632777 | 26.85301837 | 0.016036376 | 0.582363554 | 0.002296904 |
| darkorange | midnightblue | 0.000469794 | 22.54368055 |  |  |  |
| darkorange | orange | 0.000184982 | 15.01317901 | 0.011288734 | 0.417989469 | 0.00160267 |
| darkorange | plum1 |  |  | 0.005549577 | 0.219286879 | 0.047560215 |
| darkorange | purple | 0.002640196 | 79.92977219 |  |  |  |
| darkorange | salmon | 0.000321881 | 18.63282955 |  |  |  |
| darkorange | sienna3 | 7.5968E-05 | 12.13080875 |  |  |  |
| darkorange | tan | 0.002151539 | 67.00954919 | 0.008259772 | 0.313119958 | 0.040732034 |
| darkorange | white |  |  | 0.006398521 | 0.248679245 | 0.04553345 |
| darkorange | yellow | 0.004541225 | 130.1935654 |  |  |  |
| darkorange2 | red | 0.000585534 | 25.60388498 | 0.018329129 | 0.661743851 | 1.88937E-05 |
| darkorange2 | steelblue |  |  | 0.006802827 | 0.262677249 | 0.021277609 |
| darkorange2 | yellow | 0.000514451 | 23.72443484 | 0.02022935 | 0.727533793 | 2.17352E-05 |
| darkred | darkslateblue | 0.000513859 | 23.70878226 |  |  |  |
| darkred | green | 0.011799598 | 322.1071514 |  |  |  |
| darkred | grey60 | 0.001569712 | 51.62584294 | 0.03802892 | 1.343795092 | 8.30771E-11 |
| darkred | lightgreen | 0.001849323 | 59.01886487 | 0.008685833 | 0.327871149 | 0.043278117 |
| darkred | lightsteelblue1 | 0.001899602 | 60.34824642 | 0.004946148 | 0.198394816 | 0.002473631 |
| darkred | magenta | 0.000158779 | 14.32036111 |  |  |  |
| darkred | midnightblue | 0.007081445 | 197.3577589 | 0.023415061 | 0.837830306 | 6.79333E-06 |
| darkred | orange | 0.000167907 | 14.56169725 |  |  |  |
| darkred | pink | 0.001821907 | 58.2939656 | 0.048130493 | 1.693534347 | 2.0841E-11 |
| darkred | purple | 0.010237596 | 280.8073434 | 0.027223764 | 0.969696175 | 6.31028E-05 |
| darkred | salmon | 0.00343127 | 100.8460182 |  |  |  |
| darkred | sienna3 | 0.000101806 | 12.81398579 |  |  |  |
| darkred | tan | 0.005750079 | 162.1560236 |  |  |  |
| darkred | yellow | 0.024927893 | 669.2232771 | 0.041705333 | 1.471080805 | 8.34521E-06 |
| darkslateblue | green | 0.001458609 | 48.68827077 |  |  |  |
| darkslateblue | midnightblue | 0.001114217 | 39.58242275 | 0.018370384 | 0.663172199 | 4.32872E-08 |
| darkslateblue | purple | 0.001419398 | 47.65151603 | 0.019235224 | 0.693114885 | 2.84703E-09 |
| darkslateblue | salmon | 2.68967E-05 | 10.83334797 |  |  |  |
| darkslateblue | yellow | 0.00218211 | 67.81783636 | 0.019295001 | 0.695184526 | 0.006408137 |
| darkturquoise | floralwhite |  |  | 0.005045536 | 0.20183586 | 0.011833873 |
| darkturquoise | lightcyan1 |  |  | 0.004131812 | 0.170200662 | 0.045575248 |
| darkturquoise | lightgreen | 0.001262302 | 43.49783129 | 0.032926009 | 1.167120791 | 3.60214E-08 |
| darkturquoise | magenta | 5.0635E-05 | 11.46099562 |  |  |  |
| darkturquoise | orange | 0.000520023 | 23.87175678 | 0.014101408 | 0.51537059 | 9.42863E-05 |
| darkturquoise | red | 0.000153883 | 14.19090075 |  |  |  |
| darkturquoise | turquoise | 0.00196559 | 62.0929841 |  |  |  |
| floralwhite | paleturquoise |  |  | 0.004900041 | 0.196798489 | 0.004302793 |
| floralwhite | turquoise |  |  | 0.029628731 | 1.052961575 | 0.000605312 |
| green | grey60 | 0.003142748 | 93.21741915 | 0.036749765 | 1.299507855 | 1.53101E-05 |
| green | lightgreen | 0.000399615 | 20.68814643 |  |  |  |
| green | lightsteelblue1 | 0.003237202 | 95.71480859 |  |  |  |
| green | midnightblue | 0.017146695 | 463.486037 | 0.048969241 | 1.722573721 | 9.64205E-10 |
| green | purple | 0.01985574 | 535.1140035 | 0.045238733 | 1.593415105 | 3.72498E-06 |
| green | salmon | 0.002724197 | 82.15078256 |  |  |  |
| green | skyblue3 | 0.000363655 | 19.73734948 |  |  |  |
| green | tan | 0.00357397 | 104.6190431 |  |  |  |
| green | yellow | 0.047676282 | 1270.697628 | 0.052706522 | 1.851966801 | 0.001993279 |
| greenyellow | lightcyan1 | 0.000285546 | 17.67211136 | 0.010526792 | 0.391609306 | 9.16062E-05 |
| greenyellow | lightgreen | 0.003998469 | 115.8429349 | 0.036853452 | 1.303097747 | 1.09371E-13 |
| greenyellow | lightyellow | 0.000391056 | 20.46184298 | 0.011347988 | 0.420040985 | 0.049361405 |
| greenyellow | orangered4 | 0.000921206 | 34.47916378 | 0.011796537 | 0.435570747 | 0.00553958 |
| greenyellow | red | 0.003955231 | 114.6996996 |  |  |  |
| greenyellow | royalblue | 0.003795408 | 110.4739401 | 0.052451676 | 1.84314346 | 3.34377E-17 |
| greenyellow | violet | 0.000284463 | 17.64348659 | 0.007646777 | 0.291896683 | 0.016931213 |
| greenyellow | yellow | 0.001676242 | 54.44253531 |  |  |  |
| grey60 | lightsteelblue1 | 0 | 10.12219106 |  |  |  |
| grey60 | midnightblue | 0.001449403 | 48.4448358 | 0.018773759 | 0.677137942 | 0.007497108 |
| grey60 | pink | 9.2395E-05 | 12.56514264 |  |  |  |
| grey60 | purple | 0.001332692 | 45.35898492 |  |  |  |
| grey60 | yellow | 0.00327297 | 96.66051621 | 0.033251532 | 1.178391144 | 0.007932525 |
| ivory | lightcyan1 |  |  | 0.007117245 | 0.273563103 | 0.001599661 |
| lightcyan1 | lightgreen | 0.000665639 | 27.72189974 | 0.00821939 | 0.31172184 | 0.000254569 |
| lightcyan1 | orangered4 |  |  | 0.002970637 | 0.129998156 | 0.038866039 |
| lightcyan1 | red | 0.00068534 | 28.24278324 | 0.021598112 | 0.774923408 | 0.000315344 |
| lightcyan1 | violet |  |  | 0.007142601 | 0.274440998 | 0.007499898 |
| lightgreen | lightsteelblue1 | 0.00051197 | 23.6588391 | 0.009439695 | 0.353971558 | 0.000254569 |
| lightgreen | magenta | 0.002257389 | 69.80825052 | 0.023325788 | 0.834739451 | 1.2777E-05 |
| lightgreen | midnightblue | 0.000231262 | 16.23683151 |  |  |  |
| lightgreen | orange | 0.001265521 | 43.58294199 | 0.01262625 | 0.464297304 | 0.000540977 |
| lightgreen | orangered4 | 0.001694452 | 54.92403111 | 0.032738007 | 1.160611732 | 7.48164E-15 |
| lightgreen | pink | 0.001501821 | 49.83080814 | 0.024578588 | 0.878114233 | 0.001129152 |
| lightgreen | purple | 0.00609767 | 171.3464359 | 0.041264283 | 1.455810663 | 1.32592E-09 |
| lightgreen | red | 0.001177501 | 41.25568073 |  |  |  |
| lightgreen | royalblue | 0.000270299 | 17.26898367 |  |  |  |
| lightgreen | skyblue3 | 0.000147319 | 14.01736026 | 0.012667863 | 0.46573803 | 0.000135421 |
| lightgreen | turquoise | 0.004443205 | 127.6018863 |  |  |  |
| lightgreen | violet | 0.000226653 | 16.11497157 |  |  |  |
| lightgreen | white | 0.000155582 | 14.23581874 | 0.012157205 | 0.448057913 | 0.001902936 |
| lightsteelblue1 | midnightblue | 0.001325994 | 45.18188436 |  |  |  |
| lightsteelblue1 | pink |  |  | 0.007256899 | 0.278398249 | 0.027971824 |
| lightsteelblue1 | purple | 0.002692137 | 81.3031268 | 0.014987846 | 0.546061086 | 0.000477827 |
| lightsteelblue1 | royalblue |  |  | 0.004701668 | 0.189930376 | 0.011833873 |
| lightsteelblue1 | salmon | 0.000284124 | 17.63452125 |  |  |  |
| lightsteelblue1 | tan | 0.000769613 | 30.47100499 |  |  |  |
| lightsteelblue1 | yellow | 0.003995799 | 115.7723358 |  |  |  |
| lightyellow | midnightblue | 0.000753159 | 30.0359416 | 0.012579732 | 0.462686726 | 0.019566213 |
| lightyellow | royalblue | 0.000120633 | 13.31177218 |  |  |  |
| lightyellow | salmon | 0.001397584 | 47.07473085 | 0.018165253 | 0.656070081 | 0.000104997 |
| lightyellow | tan | 0.001098641 | 39.17059279 | 0.014552861 | 0.531000917 | 0.003306421 |
| lightyellow | yellow | 0.003189804 | 94.46158024 |  |  |  |
| magenta | midnightblue | 0.000175428 | 14.76056407 |  |  |  |
| magenta | orange | 0.000890585 | 33.66952884 | 0.019133348 | 0.689587721 | 6.31028E-05 |
| magenta | plum1 | 0.000501206 | 23.37423608 | 0.025350321 | 0.90483336 | 1.144E-08 |
| magenta | purple | 0.003221083 | 95.28860199 | 0.033582228 | 1.189840581 | 7.6468E-09 |
| magenta | yellow | 0.000935201 | 34.8492034 |  |  |  |
| midnightblue | plum1 | 0.000124823 | 13.42253862 |  |  |  |
| midnightblue | purple | 0.010770658 | 294.9016754 | 0.031655771 | 1.123142266 | 1.32592E-09 |
| midnightblue | salmon | 0.005083871 | 144.541289 | 0.024613098 | 0.879309027 | 1.30859E-08 |
| midnightblue | steelblue | 0.000125803 | 13.44845638 | 0.009037346 | 0.340041338 | 0.039177397 |
| midnightblue | tan | 0.002013158 | 63.35069907 |  |  |  |
| midnightblue | yellow | 0.034370038 | 918.8764719 | 0.102449986 | 3.574197853 | 1.36758E-29 |
| orange | orangered4 | 1.04628E-05 | 10.3988307 |  |  |  |
| orange | purple | 0.00030203 | 18.10796575 |  |  |  |
| orange | salmon | 0.000290538 | 17.80410921 |  |  |  |
| orange | turquoise | 0.006396084 | 179.2366138 | 0.067982753 | 2.380864429 | 8.57821E-08 |
| orangered4 | purple | 0.000223922 | 16.04274773 |  |  |  |
| orangered4 | red | 0.000493257 | 23.16405514 |  |  |  |
| orangered4 | turquoise | 0.005440868 | 153.9803961 | 0.033453806 | 1.185394305 | 8.25713E-05 |
| orangered4 | violet | 6.08523E-05 | 11.73114512 | 0.01457956 | 0.53192528 | 1.01528E-07 |
| orangered4 | yellow | 0.001347635 | 45.75408086 | 0.01822961 | 0.658298273 | 0.007721075 |
| paleturquoise | yellow | 0.000992854 | 36.37354643 | 0.032217147 | 1.142578378 | 3.81578E-05 |
| pink | purple | 0.000124797 | 13.42186769 |  |  |  |
| plum1 | purple | 0.000293533 | 17.88330385 |  |  |  |
| plum1 | steelblue |  |  | 0.010939559 | 0.405900256 | 0.000979749 |
| plum1 | yellow | 0.001122383 | 39.79832677 | 0.026753182 | 0.953403589 | 5.4902E-06 |
| purple | salmon | 0.002356163 | 72.41985065 |  |  |  |
| purple | sienna3 | 0.000111022 | 13.05764557 |  |  |  |
| purple | tan | 0.003859431 | 112.1667288 |  |  |  |
| purple | yellow | 0.026615482 | 713.843653 | 0.041692946 | 1.470651949 | 0.0020249 |
| red | royalblue | 0.001193828 | 41.68737446 |  |  |  |
| red | steelblue | 0.001524197 | 50.42242371 | 0.028191981 | 1.003218038 | 1.53911E-06 |
| red | violet | 0.000733252 | 29.50958558 | 0.016820034 | 0.609495564 | 0.006408137 |
| red | white | 0.001030773 | 37.37613187 | 0.018164397 | 0.656040458 | 0.002365364 |
| royalblue | skyblue | 9.33676E-05 | 12.59085924 | 0.012741718 | 0.468295047 | 0.04553345 |
| royalblue | steelblue | 7.16126E-06 | 10.31153687 | 0.009099281 | 0.34218567 | 0.016198916 |
| royalblue | turquoise | 0.001962686 | 62.01619886 |  |  |  |
| saddlebrown | turquoise | 0.000531448 | 24.17384028 |  |  |  |
| salmon | tan | 0.002444115 | 74.74533487 |  |  |  |
| salmon | turquoise | 0.000658539 | 27.53415833 | 0.077993668 | 2.727464914 | 2.85487E-05 |
| salmon | yellow | 0.016986453 | 459.24918 | 0.077537149 | 2.711659178 | 8.04653E-18 |
| salmon | yellowgreen | 7.87128E-05 | 12.20338092 |  |  |  |
| sienna3 | tan | 0.000149838 | 14.08394768 |  |  |  |
| sienna3 | yellow | 0.000395608 | 20.58218641 |  |  |  |
| sienna3 | yellowgreen |  |  | 0.005672619 | 0.223546887 | 0.001091721 |
| steelblue | yellow | 0.001603705 | 52.52463444 | 0.034735319 | 1.229763189 | 1.30485E-05 |
| tan | yellow | 0.019845444 | 534.8417727 | 0.053306998 | 1.872756644 | 4.46699E-11 |
| turquoise | yellowgreen | 0.003348022 | 98.64492648 | 0.028371667 | 1.009439172 | 3.92033E-06 |
| violet | yellow | 0.000871229 | 33.15774791 | 0.021118243 | 0.758309255 | 0.003302992 |

Supplement table 1 The overlapping and specific inter-module connections of *CT* and *SW*. *M_x_* and *M_y_* represent the any two different modules, respectively. *CT* and *SW* is the consistency score and sum of weight of edge between *M_x_* and *M_y_*, respectively. *CT’* and *SW’* is normalized value of *CT* and *SW*, respectively, is between 0-1. *P*-value is the *P*-value of hyper geometric distribution of direct edge connections between *M_x_* and *M_y_*. *SW* with *P*-value＜0.05 or *CT* more than 10 are shown in the table.

Supplement table 2

| **M_x_** | **M_y_** | **P-value（PS）** | **PS** | **P-value（SW）** | **SW’** | **IMCC_2_** |
| --- | --- | --- | --- | --- | --- | --- |
| blue | brown | 0 | 32.40154101 | 2.7807E-303 | 34.64940606 | 67.05094708 |
| blue | yellow | 0 | 21.98121704 | 3.5238E-114 | 13.73632105 | 35.71753809 |
| brown | yellow | 0 | 20.8086233 | 1.09561E-92 | 11.04347046 | 31.85209376 |
| blue | purple | 0 | 15.4002687 | 5.30624E-80 | 9.591037369 | 24.99130607 |
| blue | darkred | 0 | 12.74760092 | 7.99678E-76 | 8.657465877 | 21.4050668 |
| brown | purple | 0 | 15.40221582 | 3.62107E-42 | 5.52005498 | 20.9222708 |
| blue | green | 1.11022E-16 | 11.49886165 | 3.41057E-72 | 9.012745855 | 20.51160751 |
| blue | midnightblue | 0 | 12.03512532 | 2.64843E-66 | 7.383522589 | 19.4186479 |
| brown | darkred | 0 | 13.17188555 | 1.73871E-65 | 5.765834518 | 18.93772007 |
| brown | green | 0 | 12.41538632 | 6.16585E-20 | 4.696346461 | 17.11173278 |
| brown | midnightblue | 0 | 13.68772001 | 7.40254E-12 | 1.94959642 | 15.63731643 |
| midnightblue | yellow | 0 | 11.48522083 | 1.36758E-29 | 3.574197853 | 15.05941868 |
| brown | tan | 1.93166E-05 | 5.132668681 | 9.04103E-69 | 9.114277158 | 14.24694584 |
| purple | yellow | 0 | 12.090312 | 0.0020249 | 1.470651949 | 13.56096395 |
| darkred | yellow | 0 | 11.91286002 | 8.34521E-06 | 1.471080805 | 13.38394082 |
| green | yellow | 2.66454E-15 | 10.91816001 | 0.001993279 | 1.851966801 | 12.77012681 |
| blue | tan | 9.41173E-07 | 9.322540694 | 2.58579E-09 | 1.979142002 | 11.3016827 |
| darkred | purple | 0 | 9.993760529 | 6.31028E-05 | 0.969696175 | 10.9634567 |
| blue | salmon | 1.11022E-16 | 7.524414371 | 1.13338E-24 | 3.035507172 | 10.55992154 |
| brown | darkorange | 0 | 6.27885639 | 5.69485E-37 | 4.279214885 | 10.55807128 |
| blue | lightgreen | 0 | 7.735131962 | 6.71638E-14 | 2.779763654 | 10.51489562 |
| green | purple | 5.77316E-15 | 8.903313815 | 3.72498E-06 | 1.593415105 | 10.49672892 |
| blue | grey60 | 0 | 7.031010619 | 1.19042E-11 | 3.164226283 | 10.1952369 |
| black | blue | 0 | 7.141396812 | 4.11582E-09 | 2.869512267 | 10.01090908 |
| midnightblue | purple | 0 | 8.865684319 | 1.32592E-09 | 1.123142266 | 9.988826586 |
| brown | lightgreen | 0 | 7.872961922 | 2.60827E-11 | 2.049739781 | 9.922701703 |
| green | midnightblue | 0 | 8.174770774 | 9.64205E-10 | 1.722573721 | 9.897344495 |
| salmon | yellow | 0 | 7.100652613 | 8.04653E-18 | 2.711659178 | 9.81231179 |
| blue | darkorange | 0 | 7.170653914 | 2.05078E-13 | 2.543786375 | 9.714440289 |
| brown | salmon | 5.44009E-15 | 6.816582404 | 4.47799E-21 | 2.882611803 | 9.699194207 |
| brown | grey60 | 0 | 7.553782711 | 4.11253E-06 | 1.905224857 | 9.459007568 |
| blue | magenta | 0 | 6.402165828 | 4.05401E-09 | 2.869861358 | 9.272027186 |
| darkred | midnightblue | 0 | 8.423695181 | 6.79333E-06 | 0.837830306 | 9.261525487 |
| black | turquoise | 1.8614E-12 | 3.872701869 | 2.75117E-15 | 5.345720402 | 9.218422271 |
| tan | yellow | 1.44599E-07 | 7.290651746 | 4.46699E-11 | 1.872756644 | 9.16340839 |
| darkred | green | 0 | 8.748694827 |  |  | 8.748694827 |
| black | lightgreen | 0 | 5.923582321 | 7.202E-27 | 2.772496505 | 8.696078826 |
| blue | pink | 5.50286E-10 | 5.510487365 | 1.61029E-09 | 3.046396611 | 8.556883977 |
| brown | turquoise | 0 | 8.442314474 |  |  | 8.442314474 |
| blue | turquoise | 0 | 8.252343213 |  |  | 8.252343213 |
| grey60 | yellow | 0 | 6.788570891 | 0.007932525 | 1.178391144 | 7.966962035 |
| brown | magenta | 0 | 6.695285592 | 0.019696443 | 1.215949549 | 7.911235141 |
| blue | darkolivegreen | 2.68027E-06 | 5.326856724 | 4.84438E-21 | 2.55715602 | 7.884012744 |
| lightgreen | purple | 0 | 6.019127074 | 1.32592E-09 | 1.455810663 | 7.474937737 |
| black | brown | 0 | 7.234554204 |  |  | 7.234554204 |
| darkred | grey60 | 0 | 5.809000696 | 8.30771E-11 | 1.343795092 | 7.152795788 |
| brown | pink | 0 | 7.084261786 |  |  | 7.084261786 |
| turquoise | yellow | 0 | 7.006081916 |  |  | 7.006081916 |
| salmon | turquoise | 0 | 4.171279957 | 2.85487E-05 | 2.727464914 | 6.898744871 |
| green | grey60 | 7.99361E-15 | 5.58890073 | 1.53101E-05 | 1.299507855 | 6.888408584 |
| brown | darkolivegreen | 6.88141E-10 | 5.973752451 | 0.015872627 | 0.836327062 | 6.810079513 |
| darkolivegreen | green | 2.43009E-05 | 4.226115054 | 2.52138E-23 | 2.54204767 | 6.768162723 |
| brown | lightyellow | 2.44098E-06 | 3.961247467 | 2.60827E-11 | 2.648644578 | 6.609892045 |
| midnightblue | salmon | 0 | 5.721496281 | 1.30859E-08 | 0.879309027 | 6.600805308 |
| brown | brown4 | 5.16827E-11 | 4.30260379 | 7.44294E-17 | 2.235743049 | 6.538346839 |
| grey60 | midnightblue | 0 | 5.830766946 | 0.007497108 | 0.677137942 | 6.507904888 |
| black | purple | 7.11653E-14 | 5.27057831 | 6.16949E-05 | 1.156038035 | 6.426616345 |
| magenta | purple | 6.10623E-15 | 5.191731076 | 7.6468E-09 | 1.189840581 | 6.381571657 |
| blue | brown4 | 1.68187E-11 | 4.548203609 | 3.1505E-15 | 1.824626604 | 6.372830213 |
| blue | lightyellow | 1.03821E-06 | 4.954864448 | 0.002398754 | 1.329444245 | 6.284308693 |
| blue | lightsteelblue1 | 4.78984E-09 | 3.864578596 | 8.6802E-31 | 2.248282023 | 6.112860619 |
| brown | red | 0.01825656 | 1.828707753 | 9.68811E-13 | 4.182879917 | 6.01158767 |
| brown4 | yellow | 5.92859E-13 | 4.666739139 | 2.15568E-09 | 1.312027291 | 5.97876643 |
| darkorange | purple | 3.33067E-16 | 5.95896344 |  |  | 5.95896344 |
| darkorange | yellow | 3.98029E-10 | 5.925218128 |  |  | 5.925218128 |
| grey60 | purple | 0 | 5.924901585 |  |  | 5.924901585 |
| darkred | pink | 1.36815E-08 | 4.158193855 | 2.0841E-11 | 1.693534347 | 5.851728202 |
| darkorange | darkred | 1.08791E-12 | 5.246997382 | 1.92981E-05 | 0.563749672 | 5.810747054 |
| darkolivegreen | purple | 2.87207E-10 | 4.697667198 | 7.85146E-07 | 1.067430408 | 5.765097606 |
| brown | lightsteelblue1 | 3.05551E-08 | 4.081304946 | 9.27617E-22 | 1.629536668 | 5.710841613 |
| darkred | tan | 2.75226E-07 | 5.614943551 |  |  | 5.614943551 |
| brown | cyan | 1.73148E-08 | 3.553790606 | 4.67789E-09 | 1.976113322 | 5.529903928 |
| purple | turquoise | 0 | 5.477209185 |  |  | 5.477209185 |
| magenta | yellow | 2.35589E-13 | 5.396029313 |  |  | 5.396029313 |
| darkred | salmon | 4.44089E-16 | 5.350751749 |  |  | 5.350751749 |
| darkred | lightgreen | 3.3451E-13 | 4.855641032 | 0.043278117 | 0.327871149 | 5.183512181 |
| purple | salmon | 7.10543E-15 | 5.124780818 |  |  | 5.124780818 |
| black | pink | 7.07239E-08 | 3.318603297 | 3.07547E-05 | 1.801458994 | 5.120062291 |
| lightyellow | yellow | 3.49818E-10 | 5.044716968 |  |  | 5.044716968 |
| darkorange | lightgreen | 1.1724E-13 | 4.223168974 | 5.21736E-09 | 0.787153752 | 5.010322726 |
| purple | tan | 1.714E-05 | 4.970142844 |  |  | 4.970142844 |
| lightgreen | yellow | 1.33227E-15 | 4.932611533 |  |  | 4.932611533 |
| pink | purple | 1.01383E-10 | 4.923694268 |  |  | 4.923694268 |
| green | salmon | 1.43929E-12 | 4.916774607 |  |  | 4.916774607 |
| lightsteelblue1 | purple | 8.11296E-12 | 4.306955372 | 0.000477827 | 0.546061086 | 4.853016458 |
| lightgreen | turquoise | 2.66454E-15 | 4.757774178 |  |  | 4.757774178 |
| lightgreen | magenta | 1.24628E-11 | 3.902999583 | 1.2777E-05 | 0.834739451 | 4.737739034 |
| cyan | lightgreen | 9.88099E-15 | 3.872180974 | 3.52004E-07 | 0.824768575 | 4.69694955 |
| blue | darkslateblue | 0.0195804 | 2.695347061 | 7.8678E-22 | 1.99549516 | 4.690842221 |
| black | yellow | 0 | 4.658277876 |  |  | 4.658277876 |
| lightgreen | pink | 2.86166E-08 | 3.779587698 | 0.001129152 | 0.878114233 | 4.657701931 |
| midnightblue | turquoise | 0 | 4.657613518 |  |  | 4.657613518 |
| darkolivegreen | midnightblue | 6.81532E-08 | 4.163675907 | 0.014083305 | 0.491286616 | 4.654962523 |
| black | darkred | 1.31006E-14 | 4.64849804 |  |  | 4.64849804 |
| orange | turquoise | 0.000382728 | 2.23176098 | 8.57821E-08 | 2.380864429 | 4.612625409 |
| darkolivegreen | yellow | 4.24133E-06 | 4.570962844 |  |  | 4.570962844 |
| darkred | turquoise | 0 | 4.533715778 |  |  | 4.533715778 |
| brown | orange | 1.26106E-06 | 2.98829491 | 1.64021E-07 | 1.516212215 | 4.504507125 |
| green | tan | 0.000248834 | 4.446540939 |  |  | 4.446540939 |
| darkred | magenta | 9.88099E-15 | 4.445759387 |  |  | 4.445759387 |
| blue | cyan | 2.2472E-11 | 4.410140181 |  |  | 4.410140181 |
| pink | yellow | 8.45717E-08 | 4.402622619 |  |  | 4.402622619 |
| brown4 | darkred | 1.68321E-12 | 4.146765084 | 0.002473631 | 0.239250197 | 4.386015281 |
| blue | orange | 2.9752E-11 | 4.37994325 |  |  | 4.37994325 |
| darkorange | tan | 0.002470494 | 4.026059558 | 0.040732034 | 0.313119958 | 4.339179516 |
| lightyellow | salmon | 3.79136E-11 | 3.552276269 | 0.000104997 | 0.656070081 | 4.20834635 |
| blue | red | 1.02432E-06 | 4.186191622 |  |  | 4.186191622 |
| salmon | tan | 1.86141E-05 | 4.116057628 |  |  | 4.116057628 |
| magenta | turquoise | 0 | 4.113070152 |  |  | 4.113070152 |
| magenta | midnightblue | 1.67955E-12 | 4.096802851 |  |  | 4.096802851 |
| darkolivegreen | darkred | 4.19647E-10 | 4.085769396 |  |  | 4.085769396 |
| brown4 | purple | 5.66569E-11 | 4.069714601 |  |  | 4.069714601 |
| darkorange | magenta | 2.14515E-08 | 3.471504338 | 0.002296904 | 0.582363554 | 4.053867892 |
| darkslateblue | yellow | 0.002468324 | 3.276449485 | 0.006408137 | 0.695184526 | 3.971634011 |
| midnightblue | tan | 2.44323E-06 | 3.96159177 |  |  | 3.96159177 |
| darkred | lightsteelblue1 | 7.67445E-09 | 3.763090508 | 0.002473631 | 0.198394816 | 3.961485324 |
| cyan | darkorange | 4.30382E-09 | 2.996660866 | 8.61209E-08 | 0.914937315 | 3.911598181 |
| lightyellow | tan | 0.000459221 | 3.361331203 | 0.003306421 | 0.531000917 | 3.89233212 |
| brown4 | midnightblue | 2.83511E-11 | 3.633904296 | 0.025717794 | 0.209349528 | 3.843253824 |
| black | magenta | 2.29592E-10 | 3.837950837 |  |  | 3.837950837 |
| brown4 | tan | 3.71266E-07 | 3.505983949 | 0.047560215 | 0.321189904 | 3.827173853 |
| red | yellow | 1.6694E-06 | 3.817895933 |  |  | 3.817895933 |
| lightyellow | midnightblue | 5.98564E-07 | 3.342653358 | 0.019566213 | 0.462686726 | 3.805340084 |
| brown | darkslateblue | 1.475E-05 | 3.793245592 |  |  | 3.793245592 |
| darkorange | midnightblue | 8.15269E-10 | 3.792794982 |  |  | 3.792794982 |
| black | orange | 1.22072E-08 | 3.17661658 | 0.002424233 | 0.599397238 | 3.776013818 |
| lightsteelblue1 | yellow | 1.15512E-06 | 3.760435472 |  |  | 3.760435472 |
| darkslateblue | midnightblue | 0.000199571 | 3.091348708 | 4.32872E-08 | 0.663172199 | 3.754520906 |
| midnightblue | pink | 3.30291E-07 | 3.726374785 |  |  | 3.726374785 |
| darkorange | green | 0.000902465 | 3.724210931 |  |  | 3.724210931 |
| darkslateblue | purple | 0.000173386 | 3.004682918 | 2.84703E-09 | 0.693114885 | 3.697797803 |
| darkred | lightyellow | 1.14794E-08 | 3.691307444 |  |  | 3.691307444 |
| darkolivegreen | grey60 | 1.5276E-07 | 3.348827336 | 0.004445994 | 0.332520947 | 3.681348283 |
| darkturquoise | lightgreen | 2.0033E-09 | 2.491415649 | 3.60214E-08 | 1.167120791 | 3.658536439 |
| green | pink | 0.000174085 | 3.632828636 |  |  | 3.632828636 |
| brown4 | green | 7.92546E-07 | 3.626077778 |  |  | 3.626077778 |
| green | turquoise | 2.1718E-10 | 3.606274468 |  |  | 3.606274468 |
| grey60 | salmon | 9.87253E-11 | 3.518951892 |  |  | 3.518951892 |
| black | midnightblue | 2.97445E-10 | 3.498860303 |  |  | 3.498860303 |
| black | darkorange | 1.22072E-08 | 3.434375351 |  |  | 3.434375351 |
| lightgreen | lightsteelblue1 | 4.75556E-10 | 3.073182008 | 0.000254569 | 0.353971558 | 3.427153566 |
| blue | plum1 | 0.000574335 | 2.643958303 | 0.001693134 | 0.775378401 | 3.419336704 |
| paleturquoise | yellow | 6.63022E-05 | 2.250288264 | 3.81578E-05 | 1.142578378 | 3.392866642 |
| green | lightsteelblue1 | 0.001087818 | 3.391686565 |  |  | 3.391686565 |
| blue | skyblue | 5.10382E-06 | 2.228426681 | 0.005625076 | 1.161161389 | 3.38958807 |
| green | lightyellow | 2.44507E-06 | 3.348558998 |  |  | 3.348558998 |
| lightgreen | midnightblue | 1.12563E-10 | 3.326227589 |  |  | 3.326227589 |
| green | lightgreen | 2.06191E-05 | 3.321497894 |  |  | 3.321497894 |
| brown | skyblue3 | 0.000187164 | 2.404587278 | 0.000383027 | 0.872258721 | 3.276845999 |
| lightyellow | purple | 4.9209E-07 | 3.252803658 |  |  | 3.252803658 |
| green | magenta | 1.98002E-07 | 3.210160126 |  |  | 3.210160126 |
| blue | paleturquoise | 0.001636645 | 2.417408773 | 0.043059035 | 0.792130483 | 3.209539256 |
| pink | turquoise | 2.12527E-09 | 3.181841471 |  |  | 3.181841471 |
| magenta | salmon | 1.95373E-10 | 3.173798553 |  |  | 3.173798553 |
| cyan | purple | 2.02965E-07 | 3.165595438 |  |  | 3.165595438 |
| lightgreen | orangered4 | 0.01800937 | 2.00496094 | 7.48164E-15 | 1.160611732 | 3.165572672 |
| grey60 | pink | 1.33397E-05 | 3.164489722 |  |  | 3.164489722 |
| black | lightsteelblue1 | 2.02965E-07 | 2.631318417 | 0.000540977 | 0.520468804 | 3.151787221 |
| lightgreen | orange | 2.67607E-08 | 2.658779652 | 0.000540977 | 0.464297304 | 3.123076956 |
| darkorange | salmon | 1.10876E-05 | 3.115357522 |  |  | 3.115357522 |
| blue | skyblue3 | 9.28416E-05 | 2.413224212 | 0.016724637 | 0.701366816 | 3.114591028 |
| lightsteelblue1 | midnightblue | 1.85341E-06 | 3.109561686 |  |  | 3.109561686 |
| darkorange | turquoise | 6.15597E-12 | 3.081344079 |  |  | 3.081344079 |
| grey60 | tan | 7.48924E-05 | 3.072524286 |  |  | 3.072524286 |
| brown4 | salmon | 1.69242E-07 | 3.058579798 |  |  | 3.058579798 |
| steelblue | yellow | 0.03310703 | 1.827373722 | 1.30485E-05 | 1.229763189 | 3.057136912 |
| brown4 | grey60 | 1.88923E-10 | 3.041709526 |  |  | 3.041709526 |
| black | green | 0.00016184 | 3.03478007 |  |  | 3.03478007 |
| brown4 | darkorange | 9.34305E-08 | 3.00896479 |  |  | 3.00896479 |
| plum1 | yellow | 0.00079499 | 2.041210769 | 5.4902E-06 | 0.953403589 | 2.994614358 |
| darkorange | grey60 | 6.59586E-07 | 2.986936621 |  |  | 2.986936621 |
| darkgreen | lightgreen | 2.93008E-06 | 2.272338131 | 6.79333E-06 | 0.709878603 | 2.982216734 |
| lightyellow | turquoise | 7.12933E-10 | 2.971642303 |  |  | 2.971642303 |
| darkred | darkslateblue | 3.26925E-05 | 2.935251657 |  |  | 2.935251657 |
| lightsteelblue1 | pink | 1.05731E-06 | 2.653612491 | 0.027971824 | 0.278398249 | 2.93201074 |
| magenta | orange | 3.92892E-05 | 2.217480675 | 6.31028E-05 | 0.689587721 | 2.907068396 |
| cyan | darkred | 2.67634E-06 | 2.887848805 |  |  | 2.887848805 |
| darkslateblue | green | 0.005045062 | 2.852983261 |  |  | 2.852983261 |
| black | darkturquoise | 2.86166E-08 | 2.839121465 |  |  | 2.839121465 |
| grey60 | turquoise | 2.28373E-13 | 2.825196881 |  |  | 2.825196881 |
| darkorange | lightsteelblue1 | 4.18693E-06 | 2.801906136 |  |  | 2.801906136 |
| darkolivegreen | lightsteelblue1 | 0.000410711 | 2.445115978 | 8.4515E-05 | 0.349545504 | 2.794661482 |
| black | salmon | 2.69642E-07 | 2.781913278 |  |  | 2.781913278 |
| brown | darkgrey | 0.02699696 | 1.438433683 | 1.72502E-08 | 1.313291749 | 2.751725432 |
| orange | yellow | 3.96388E-07 | 2.734872433 |  |  | 2.734872433 |
| lightgreen | red | 0.00004365 | 2.725239526 |  |  | 2.725239526 |
| darkolivegreen | pink | 3.09707E-05 | 2.704027084 |  |  | 2.704027084 |
| brown | skyblue | 5.81832E-07 | 2.691684682 |  |  | 2.691684682 |
| brown | plum1 | 0.000100143 | 2.658802248 |  |  | 2.658802248 |
| tan | turquoise | 9.57492E-05 | 2.626928351 |  |  | 2.626928351 |
| magenta | plum1 | 0.000523526 | 1.693111294 | 1.144E-08 | 0.90483336 | 2.597944654 |
| darkorange | red | 4.72212E-05 | 2.59657093 |  |  | 2.59657093 |
| black | cyan | 2.69642E-07 | 2.558993946 |  |  | 2.558993946 |
| darkorange | pink | 5.51794E-05 | 2.547948138 |  |  | 2.547948138 |
| grey60 | lightsteelblue1 | 1.54766E-05 | 2.538491749 |  |  | 2.538491749 |
| brown | steelblue | 2.9413E-06 | 2.536355258 |  |  | 2.536355258 |
| darkorange | orange | 2.57595E-05 | 2.115860616 | 0.00160267 | 0.417989469 | 2.533850085 |
| blue | darkturquoise | 1.04601E-05 | 2.514831121 |  |  | 2.514831121 |
| magenta | pink | 1.12569E-05 | 2.510168916 |  |  | 2.510168916 |
| orange | purple | 2.02046E-05 | 2.502658831 |  |  | 2.502658831 |
| purple | skyblue | 2.48802E-07 | 2.484672827 |  |  | 2.484672827 |
| grey60 | lightyellow | 1.10876E-05 | 2.470793747 |  |  | 2.470793747 |
| brown4 | lightyellow | 7.77596E-06 | 2.468428067 |  |  | 2.468428067 |
| darkolivegreen | lightgreen | 0.000314425 | 2.457373725 |  |  | 2.457373725 |
| brown | paleturquoise | 2.48074E-05 | 2.449269319 |  |  | 2.449269319 |
| darkred | red | 0.0114482 | 2.443461464 |  |  | 2.443461464 |
| cyan | yellow | 0.000187164 | 2.441638301 |  |  | 2.441638301 |
| darkorange | lightyellow | 0.000523526 | 2.424102408 |  |  | 2.424102408 |
| brown | darkturquoise | 8.60281E-05 | 2.418643373 |  |  | 2.418643373 |
| lightgreen | skyblue3 | 5.35775E-05 | 1.941641194 | 0.000135421 | 0.46573803 | 2.407379224 |
| midnightblue | paleturquoise | 9.36882E-05 | 2.377734576 |  |  | 2.377734576 |
| grey60 | magenta | 2.2292E-06 | 2.360708044 |  |  | 2.360708044 |
| purple | red | 0.003861873 | 2.351728921 |  |  | 2.351728921 |
| darkred | orange | 0.000173386 | 2.34733921 |  |  | 2.34733921 |
| black | darkolivegreen | 0.000351393 | 2.342419314 |  |  | 2.342419314 |
| grey60 | lightgreen | 4.24889E-05 | 2.331528774 |  |  | 2.331528774 |
| floralwhite | turquoise | 0.001449487 | 1.277394369 | 0.000605312 | 1.052961575 | 2.330355943 |
| cyan | red | 1.03325E-05 | 2.322824009 |  |  | 2.322824009 |
| skyblue | yellow | 8.64024E-07 | 2.311794388 |  |  | 2.311794388 |
| brown | greenyellow | 3.51653E-05 | 2.294289052 |  |  | 2.294289052 |
| darkred | skyblue | 1.5276E-07 | 2.274156079 |  |  | 2.274156079 |
| blue | steelblue | 0.02699696 | 2.273644651 |  |  | 2.273644651 |
| brown4 | turquoise | 4.75733E-07 | 2.265275578 |  |  | 2.265275578 |
| lightgreen | salmon | 5.03736E-06 | 2.264131085 |  |  | 2.264131085 |
| darkturquoise | orange | 0.001656265 | 1.747903069 | 9.42863E-05 | 0.51537059 | 2.263273659 |
| darkslateblue | grey60 | 0.001860415 | 2.262602607 |  |  | 2.262602607 |
| purple | skyblue3 | 0.000012061 | 2.251193215 |  |  | 2.251193215 |
| orange | salmon | 0.000274355 | 2.249354464 |  |  | 2.249354464 |
| plum1 | purple | 2.37682E-05 | 2.245775449 |  |  | 2.245775449 |
| cyan | darkturquoise | 0.001472006 | 1.616487647 | 0.002132851 | 0.620466299 | 2.236953946 |
| lightsteelblue1 | magenta | 4.96167E-05 | 2.235568256 |  |  | 2.235568256 |
| midnightblue | plum1 | 3.50634E-06 | 2.225901726 |  |  | 2.225901726 |
| midnightblue | skyblue | 6.59586E-07 | 2.224193843 |  |  | 2.224193843 |
| midnightblue | steelblue | 0.02579117 | 1.869941557 | 0.039177397 | 0.340041338 | 2.209982895 |
| skyblue | turquoise | 3.1644E-10 | 2.203705665 |  |  | 2.203705665 |
| lightsteelblue1 | turquoise | 9.4268E-08 | 2.198346114 |  |  | 2.198346114 |
| cyan | magenta | 3.35438E-05 | 2.196049559 |  |  | 2.196049559 |
| lightgreen | tan | 0.01509248 | 2.163974964 |  |  | 2.163974964 |
| brown4 | magenta | 2.57595E-05 | 2.161033813 |  |  | 2.161033813 |
| red | white | 0.004539226 | 1.491527139 | 0.002365364 | 0.656040458 | 2.147567597 |
| brown | darkgreen | 2.28349E-05 | 2.145812948 |  |  | 2.145812948 |
| lightyellow | magenta | 4.61103E-06 | 2.145353693 |  |  | 2.145353693 |
| black | grey60 | 6.06129E-06 | 2.138936809 |  |  | 2.138936809 |
| magenta | tan | 0.03104445 | 2.120841853 |  |  | 2.120841853 |
| darkgreen | orangered4 | 0.01236619 | 1.51347971 | 2.42733E-05 | 0.588274821 | 2.101754531 |
| darkolivegreen | salmon | 0.01075601 | 2.096900826 |  |  | 2.096900826 |
| cyan | turquoise | 5.4432E-08 | 2.091763931 |  |  | 2.091763931 |
| green | skyblue | 1.12569E-05 | 2.086517217 |  |  | 2.086517217 |
| darkred | paleturquoise | 0.001033513 | 2.073763351 |  |  | 2.073763351 |
| brown4 | lightsteelblue1 | 0.003827359 | 2.05729184 |  |  | 2.05729184 |
| lightsteelblue1 | salmon | 0.007574825 | 2.051536905 |  |  | 2.051536905 |
| darkslateblue | salmon | 0.001755746 | 2.04596602 |  |  | 2.04596602 |
| black | darkgreen | 1.53627E-06 | 2.042061046 |  |  | 2.042061046 |
| brown4 | lightgreen | 0.000359684 | 2.035875741 |  |  | 2.035875741 |
| pink | salmon | 0.000960878 | 2.027721953 |  |  | 2.027721953 |
| lightyellow | red | 0.002612954 | 2.014142406 |  |  | 2.014142406 |
| darkolivegreen | magenta | 0.000858637 | 2.012678086 |  |  | 2.012678086 |
| lightgreen | white | 0.000913768 | 1.563336489 | 0.001902936 | 0.448057913 | 2.011394401 |
| green | paleturquoise | 0.001647672 | 1.99646018 |  |  | 1.99646018 |
| paleturquoise | purple | 0.001472006 | 1.995185733 |  |  | 1.995185733 |
| cyan | lightsteelblue1 | 1.69022E-06 | 1.992713245 |  |  | 1.992713245 |
| red | salmon | 0.006587683 | 1.969776123 |  |  | 1.969776123 |
| brown4 | darkolivegreen | 0.01354596 | 1.966626234 |  |  | 1.966626234 |
| paleturquoise | salmon | 0.001561755 | 1.965069064 |  |  | 1.965069064 |
| darkred | plum1 | 0.000108509 | 1.961847439 |  |  | 1.961847439 |
| darkolivegreen | darkslateblue | 0.02048239 | 1.958835772 |  |  | 1.958835772 |
| darkred | skyblue3 | 0.00109843 | 1.943669516 |  |  | 1.943669516 |
| salmon | skyblue | 2.67634E-06 | 1.923668522 |  |  | 1.923668522 |
| cyan | orange | 0.000274355 | 1.923424783 |  |  | 1.923424783 |
| darkolivegreen | turquoise | 0.000410014 | 1.921161685 |  |  | 1.921161685 |
| darkslateblue | pink | 0.009323329 | 1.920371263 |  |  | 1.920371263 |
| cyan | white | 0.001472006 | 1.405517273 | 0.000656425 | 0.509637618 | 1.91515489 |
| darkslateblue | magenta | 0.001656265 | 1.91272255 |  |  | 1.91272255 |
| cyan | darkgreen | 0.006523509 | 1.475639752 | 0.040732034 | 0.421130595 | 1.896770346 |
| darkgrey | darkorange | 0.000314425 | 1.88972141 |  |  | 1.88972141 |
| darkslateblue | turquoise | 0.000469869 | 1.887005005 |  |  | 1.887005005 |
| darkgrey | darkred | 0.000558601 | 1.882259805 |  |  | 1.882259805 |
| blue | darkgrey | 0.006998113 | 1.878488597 |  |  | 1.878488597 |
| greenyellow | turquoise | 0.000995754 | 1.864396755 |  |  | 1.864396755 |
| cyan | pink | 0.01757964 | 1.853764123 |  |  | 1.853764123 |
| magenta | skyblue | 5.03736E-06 | 1.846270591 |  |  | 1.846270591 |
| greenyellow | royalblue |  |  | 3.34377E-17 | 1.84314346 | 1.84314346 |
| blue | greenyellow | 0.00598912 | 1.83369996 |  |  | 1.83369996 |
| brown4 | pink | 0.01442684 | 1.816946239 |  |  | 1.816946239 |
| darkgrey | lightgreen | 8.69923E-05 | 1.802153816 |  |  | 1.802153816 |
| skyblue3 | yellow | 0.0232737 | 1.791468453 |  |  | 1.791468453 |
| midnightblue | orange | 0.000913768 | 1.789589353 |  |  | 1.789589353 |
| darkgrey | yellow | 0.02136352 | 1.763618664 |  |  | 1.763618664 |
| grey60 | paleturquoise | 0.000558601 | 1.761656341 |  |  | 1.761656341 |
| red | turquoise | 0.000201302 | 1.75885592 |  |  | 1.75885592 |
| lightgreen | lightyellow | 0.002470494 | 1.756507341 |  |  | 1.756507341 |
| darkgrey | purple | 0.006201655 | 1.74935417 |  |  | 1.74935417 |
| blue | darkgreen | 0.01107608 | 1.748813863 |  |  | 1.748813863 |
| darkorange | skyblue3 | 0.005007777 | 1.719884317 |  |  | 1.719884317 |
| grey60 | skyblue | 8.69923E-05 | 1.707598731 |  |  | 1.707598731 |
| brown4 | red | 0.02982514 | 1.702109772 |  |  | 1.702109772 |
| lightsteelblue1 | lightyellow | 0.001860415 | 1.69458457 |  |  | 1.69458457 |
| darkturquoise | purple | 0.009782105 | 1.682062254 |  |  | 1.682062254 |
| darkturquoise | magenta | 0.02277271 | 1.674693706 |  |  | 1.674693706 |
| plum1 | turquoise | 0.000241144 | 1.671288426 |  |  | 1.671288426 |
| darkorange | plum1 | 0.01880543 | 1.447443888 | 0.047560215 | 0.219286879 | 1.666730767 |
| darkturquoise | turquoise | 0.005681383 | 1.65876814 |  |  | 1.65876814 |
| blue | floralwhite | 0.003094821 | 1.656661231 |  |  | 1.656661231 |
| lightyellow | skyblue | 4.59261E-05 | 1.65313272 |  |  | 1.65313272 |
| black | red | 0.01873468 | 1.649396425 |  |  | 1.649396425 |
| blue | white | 0.01713127 | 1.637570526 |  |  | 1.637570526 |
| darkorange | white | 0.03773055 | 1.369299146 | 0.04553345 | 0.248679245 | 1.617978391 |
| cyan | darkgrey | 8.07357E-05 | 1.616912644 |  |  | 1.616912644 |
| plum1 | salmon | 0.000314425 | 1.613330356 |  |  | 1.613330356 |
| black | lightyellow | 0.001647672 | 1.61326831 |  |  | 1.61326831 |
| darkorange | skyblue | 0.001656265 | 1.600081664 |  |  | 1.600081664 |
| paleturquoise | turquoise | 0.009567909 | 1.591401208 |  |  | 1.591401208 |
| floralwhite | midnightblue | 5.78259E-05 | 1.586947874 |  |  | 1.586947874 |
| black | brown4 | 0.008882602 | 1.586391579 |  |  | 1.586391579 |
| orange | red | 0.002764867 | 1.586338883 |  |  | 1.586338883 |
| floralwhite | yellow | 0.000266872 | 1.584329651 |  |  | 1.584329651 |
| brown | floralwhite | 0.001144603 | 1.580919257 |  |  | 1.580919257 |
| midnightblue | red | 0.01091806 | 1.57333585 |  |  | 1.57333585 |
| lightyellow | orange | 0.02475189 | 1.572446675 |  |  | 1.572446675 |
| darkgrey | red | 0.000960878 | 1.563029805 |  |  | 1.563029805 |
| cyan | skyblue3 | 0.004511717 | 1.539963509 |  |  | 1.539963509 |
| brown4 | skyblue | 0.003257894 | 1.527417594 |  |  | 1.527417594 |
| brown4 | cyan | 0.01800937 | 1.527040739 |  |  | 1.527040739 |
| pink | skyblue | 0.001087818 | 1.526061589 |  |  | 1.526061589 |
| darkorange | darkturquoise | 0.02579117 | 1.525374914 |  |  | 1.525374914 |
| steelblue | turquoise | 0.002191246 | 1.521887074 |  |  | 1.521887074 |
| black | skyblue3 | 0.005315462 | 1.518751244 |  |  | 1.518751244 |
| floralwhite | salmon | 0.000336371 | 1.518182555 |  |  | 1.518182555 |
| green | plum1 | 0.01199939 | 1.504470254 |  |  | 1.504470254 |
| black | skyblue | 0.004786453 | 1.499225234 |  |  | 1.499225234 |
| lightgreen | skyblue | 0.006859344 | 1.495078901 |  |  | 1.495078901 |
| lightyellow | pink | 0.02749865 | 1.489700902 |  |  | 1.489700902 |
| cyan | midnightblue | 0.03359883 | 1.488516317 |  |  | 1.488516317 |
| lightsteelblue1 | skyblue3 | 0.009194612 | 1.435683916 |  |  | 1.435683916 |
| magenta | paleturquoise | 0.006523509 | 1.431946826 |  |  | 1.431946826 |
| darkgreen | turquoise | 0.002065469 | 1.409100932 |  |  | 1.409100932 |
| black | floralwhite | 0.008882602 | 1.383103292 |  |  | 1.383103292 |
| magenta | steelblue | 0.01025948 | 1.381186849 |  |  | 1.381186849 |
| grey60 | plum1 | 0.004754233 | 1.366077505 |  |  | 1.366077505 |
| saddlebrown | yellow | 0.009447986 | 1.354303981 |  |  | 1.354303981 |
| lightsteelblue1 | skyblue | 0.005272743 | 1.352573962 |  |  | 1.352573962 |
| floralwhite | magenta | 0.000314425 | 1.351742684 |  |  | 1.351742684 |
| lightyellow | paleturquoise | 0.02579117 | 1.337407098 |  |  | 1.337407098 |
| darkslateblue | skyblue | 0.04223942 | 1.319938504 |  |  | 1.319938504 |
| darkgreen | darkturquoise | 0.00097201 | 1.308909115 |  |  | 1.308909115 |
| greenyellow | lightgreen |  |  | 1.09371E-13 | 1.303097747 | 1.303097747 |
| cyan | lightyellow | 0.0484651 | 1.300730739 |  |  | 1.300730739 |
| darkolivegreen | skyblue | 0.03919068 | 1.289066747 |  |  | 1.289066747 |
| brown4 | plum1 | 0.02136462 | 1.285592095 |  |  | 1.285592095 |
| darkgrey | magenta | 0.01509248 | 1.276882924 |  |  | 1.276882924 |
| darkred | floralwhite | 0.008351926 | 1.245130205 |  |  | 1.245130205 |
| floralwhite | purple | 0.0172407 | 1.225475697 |  |  | 1.225475697 |
| black | white | 0.02749865 | 1.222261261 |  |  | 1.222261261 |
| darkgrey | lightsteelblue1 | 0.01962955 | 1.213880971 |  |  | 1.213880971 |
| orangered4 | turquoise |  |  | 8.25713E-05 | 1.185394305 | 1.185394305 |
| plum1 | skyblue | 0.02797265 | 1.181056666 |  |  | 1.181056666 |
| saddlebrown | salmon | 0.004279846 | 1.169126731 |  |  | 1.169126731 |
| black | darkgrey | 0.02749865 | 1.164475539 |  |  | 1.164475539 |
| brown | sienna3 |  |  | 9.92252E-09 | 1.131605354 | 1.131605354 |
| darkgrey | skyblue3 | 0.01236619 | 1.104026663 |  |  | 1.104026663 |
| midnightblue | saddlebrown | 0.02579117 | 1.01491773 |  |  | 1.01491773 |
| turquoise | yellowgreen |  |  | 3.92033E-06 | 1.009439172 | 1.009439172 |
| red | steelblue |  |  | 1.53911E-06 | 1.003218038 | 1.003218038 |
| floralwhite | lightgreen | 0.0406934 | 0.997447742 |  |  | 0.997447742 |
| brown | white |  |  | 0.035135175 | 0.88855897 | 0.88855897 |
| black | orangered4 |  |  | 1.30859E-08 | 0.784985612 | 0.784985612 |
| lightcyan1 | red |  |  | 0.000315344 | 0.774923408 | 0.774923408 |
| darkgreen | red |  |  | 0.000971031 | 0.768708966 | 0.768708966 |
| violet | yellow |  |  | 0.003302992 | 0.758309255 | 0.758309255 |
| darkorange2 | yellow |  |  | 2.17352E-05 | 0.727533793 | 0.727533793 |
| darkorange2 | red |  |  | 1.88937E-05 | 0.661743851 | 0.661743851 |
| orangered4 | yellow |  |  | 0.007721075 | 0.658298273 | 0.658298273 |
| red | violet |  |  | 0.006408137 | 0.609495564 | 0.609495564 |
| cyan | lightcyan1 |  |  | 3.28936E-06 | 0.562747518 | 0.562747518 |
| orangered4 | violet |  |  | 1.01528E-07 | 0.53192528 | 0.53192528 |
| darkgreen | violet |  |  | 0.003774427 | 0.495328945 | 0.495328945 |
| darkgreen | greenyellow |  |  | 0.016373892 | 0.488721688 | 0.488721688 |
| royalblue | skyblue |  |  | 0.04553345 | 0.468295047 | 0.468295047 |
| greenyellow | orangered4 |  |  | 0.00553958 | 0.435570747 | 0.435570747 |
| greenyellow | lightyellow |  |  | 0.049361405 | 0.420040985 | 0.420040985 |
| darkgreen | lightcyan1 |  |  | 0.000419454 | 0.412174283 | 0.412174283 |
| plum1 | steelblue |  |  | 0.000979749 | 0.405900256 | 0.405900256 |
| bisque4 | red |  |  | 0.003585366 | 0.402955909 | 0.402955909 |
| greenyellow | lightcyan1 |  |  | 9.16062E-05 | 0.391609306 | 0.391609306 |
| royalblue | steelblue |  |  | 0.016198916 | 0.34218567 | 0.34218567 |
| darkgreen | skyblue3 |  |  | 0.003774427 | 0.316168606 | 0.316168606 |
| lightcyan1 | lightgreen |  |  | 0.000254569 | 0.31172184 | 0.31172184 |
| black | lightcyan1 |  |  | 0.006022613 | 0.305297553 | 0.305297553 |
| greenyellow | violet |  |  | 0.016931213 | 0.291896683 | 0.291896683 |
| lightcyan1 | violet |  |  | 0.007499898 | 0.274440998 | 0.274440998 |
| ivory | lightcyan1 |  |  | 0.001599661 | 0.273563103 | 0.273563103 |
| darkorange2 | steelblue |  |  | 0.021277609 | 0.262677249 | 0.262677249 |
| bisque4 | greenyellow |  |  | 0.02775 | 0.24810199 | 0.24810199 |
| bisque4 | lightgreen |  |  | 0.00665549 | 0.228652375 | 0.228652375 |
| sienna3 | yellowgreen |  |  | 0.001091721 | 0.223546887 | 0.223546887 |
| darkturquoise | floralwhite |  |  | 0.011833873 | 0.20183586 | 0.20183586 |
| floralwhite | paleturquoise |  |  | 0.004302793 | 0.196798489 | 0.196798489 |
| lightsteelblue1 | royalblue |  |  | 0.011833873 | 0.189930376 | 0.189930376 |
| darkturquoise | lightcyan1 |  |  | 0.045575248 | 0.170200662 | 0.170200662 |
| bisque4 | yellowgreen |  |  | 0.038866039 | 0.161948159 | 0.161948159 |
| lightcyan1 | orangered4 |  |  | 0.038866039 | 0.129998156 | 0.129998156 |

Supplement table 2 The overlapping and specific inter-module connections of *PS* and *SW*. *PS* is the inter-module path strength of *M_x_* and *M_y_*. P-value of hyper geometric distribution of direct edge connections (*P*-value for *SW*) and indirect path connections (*P*-value for *PS*) between *M_x_* and *M_y_*. *SW* and *PS* with *P*-value＜0.05 are shown in the table.

Supplement table 3

| **M_x_** | **M_y_** | **IMCC_1_=α·SW+β·CT** | | | | | | | | | **JS** |
| --- | --- | --- | --- | --- | --- | --- | --- | --- | --- | --- | --- |
|  |  | **ρ=1/10** | **ρ=1/8** | **ρ=1/4** | **ρ=1/2** | **ρ=1/1** | **ρ=2/1** | **ρ=4/1** | **ρ=8/1** | **ρ=10/1** |  |
| blue | brown | 1 | 1 | 1 | 1 | 1 | 1 | 1 | 1 | 1 | 0.33333 |
| blue | yellow | 0.363325313 | 0.364050621 | 0.367241974 | 0.372029004 | 0.378012791 | 0.383996578 | 0.388783607 | 0.39197496 | 0.392700268 | 0.857142857 |
| blue | green | 0.158411579 | 0.160658709 | 0.170546078 | 0.185377132 | 0.20391595 | 0.222454768 | 0.237285822 | 0.247173192 | 0.249420321 | 0.833333333 |
| blue | midnightblue | 0.074471531 | 0.077538282 | 0.091031983 | 0.111272536 | 0.136573226 | 0.161873917 | 0.182114469 | 0.195608171 | 0.198674921 | 0.75 |
| brown | green | 0.100081432 | 0.100854315 | 0.104255001 | 0.109356031 | 0.115732317 | 0.122108604 | 0.127209633 | 0.130610319 | 0.131383202 | 0.33333 |
| black | turquoise | 0.056162747 | 0.058328401 | 0.067857275 | 0.082150586 | 0.100017226 | 0.117883865 | 0.132177177 | 0.141706051 | 0.143871704 | 0.833333333 |
| brown | darkorange | 0.025724719 | 0.027882239 | 0.037375325 | 0.051614954 | 0.069414491 | 0.087214028 | 0.101453657 | 0.110946743 | 0.113104263 | 0.6 |
| midnightblue | yellow | 0.040559124 | 0.041934476 | 0.047986027 | 0.057063354 | 0.068410012 | 0.07975667 | 0.088833996 | 0.094885547 | 0.0962609 | 1 |
| blue | salmon | 0.029921878 | 0.031187857 | 0.036758161 | 0.045113617 | 0.055557938 | 0.066002259 | 0.074357715 | 0.07992802 | 0.081193998 | 0.5 |
| black | blue | 0.033161451 | 0.034248895 | 0.03903365 | 0.046210783 | 0.055182198 | 0.064153613 | 0.071330745 | 0.0761155 | 0.077202944 | 0.714285714 |
| brown | salmon | 0.026974893 | 0.028208224 | 0.033634882 | 0.041774868 | 0.051949851 | 0.062124834 | 0.07026482 | 0.075691478 | 0.076924809 | 0.5 |
| green | yellow | 0.048133577 | 0.048235198 | 0.04868233 | 0.049353029 | 0.050191402 | 0.051029775 | 0.051700474 | 0.052147606 | 0.052249227 | 0.714285714 |
| blue | grey60 | 0.0163246 | 0.017975358 | 0.025238694 | 0.036133697 | 0.049752451 | 0.063371205 | 0.074266209 | 0.081529544 | 0.083180303 | 0.625 |
| salmon | yellow | 0.022491061 | 0.023714308 | 0.029096592 | 0.037170018 | 0.047261801 | 0.057353583 | 0.065427009 | 0.070809294 | 0.07203254 | 0.714285714 |
| blue | darkorange | 0.019226689 | 0.020414729 | 0.025642101 | 0.03348316 | 0.043284483 | 0.053085806 | 0.060926865 | 0.066154237 | 0.067342276 | 0.6 |
| salmon | turquoise | 0.007689005 | 0.009251331 | 0.016125565 | 0.026436915 | 0.039326104 | 0.052215292 | 0.062526642 | 0.069400876 | 0.070963202 | 0.727272727 |
| green | midnightblue | 0.020039654 | 0.020682534 | 0.023511204 | 0.02775421 | 0.033057968 | 0.038361726 | 0.042604732 | 0.045433403 | 0.046076283 | 0.5 |
| brown | cyan | 0.010390064 | 0.011410112 | 0.015898328 | 0.022630651 | 0.031046054 | 0.039461458 | 0.04619378 | 0.050681996 | 0.051702045 | 0.5 |
| green | grey60 | 0.006197932 | 0.006876861 | 0.009864152 | 0.014345087 | 0.019946257 | 0.025547426 | 0.030028362 | 0.033015652 | 0.033694582 | 0.5 |
| grey60 | yellow | 0.005998294 | 0.006603921 | 0.009268682 | 0.013265824 | 0.018262251 | 0.023258678 | 0.02725582 | 0.029920581 | 0.030526209 | 0.555555556 |
| midnightblue | salmon | 0.006859255 | 0.007253785 | 0.008989716 | 0.011593613 | 0.014848484 | 0.018103355 | 0.020707252 | 0.022443184 | 0.022837713 | 0.5 |

Supplement table 3 Jaccard Similarity (JS) coefficient of based on KEGG versus *IMCC_1_* value of top 21 module pair. The three highlighted module pair with red letter were removed as outliers in the curve fitting method.

Supplement table 4

| **Num** | **M_x_** | **M_y_** | **Average characteristic path length of inter-module** | **IMCC** | **-log2 of IMCC** |
| --- | --- | --- | --- | --- | --- |
| 1 | blue | brown | 1.238333333 | 1 | 0 |
| 2 | blue | yellow | 1.474 | 0.378012791 | 1.426512996 |
| 3 | brown | yellow | 1.5125 | 0.316319552 | 1.66338591 |
| 4 | blue | green | 1.5425 | 0.20391595 | 2.431464806 |
| 5 | blue | purple | 1.364444444 | 0.177571915 | 2.789021465 |
| 6 | blue | darkred | 1.24 | 0.154283967 | 3.027713902 |
| 7 | brown | tan | 1.369791667 | 0.152460294 | 3.11039156 |
| 8 | blue | midnightblue | 1.337142857 | 0.136573226 | 3.167830544 |
| 9 | brown | green | 1.7421875 | 0.115732317 | 3.192895313 |
| 10 | brown | purple | 1.560185185 | 0.11029046 | 3.408591911 |
| 11 | brown | darkred | 1.277777778 | 0.109286304 | 3.466502559 |
| 12 | black | turquoise | 1.820197044 | 0.100017226 | 3.605585315 |
| 13 | midnightblue | yellow | 1.5 | 0.068410012 | 4.131291649 |
| 14 | brown | darkorange | 1.465277778 | 0.069414491 | 4.276067069 |
| 15 | brown | red | 1.775 | 0.068661278 | 4.27821526 |
| 16 | green | yellow | 1.875 | 0.050191402 | 4.340717572 |
| 17 | brown | midnightblue | 1.69047619 | 0.049095827 | 4.412660003 |
| 18 | black | blue | 1.814285714 | 0.055182198 | 4.43562667 |
| 19 | blue | salmon | 1.635 | 0.055557938 | 4.470293216 |
| 20 | blue | lightgreen | 1.674285714 | 0.051581561 | 4.564085316 |
| 21 | brown | salmon | 1.65625 | 0.051949851 | 4.581220921 |
| 22 | blue | tan | 1.83 | 0.043837879 | 4.656271291 |
| 23 | salmon | yellow | 1.6375 | 0.047261801 | 4.749716803 |
| 24 | blue | darkolivegreen | 1.552 | 0.045612669 | 4.777593532 |
| 25 | blue | grey60 | 1.754285714 | 0.049752451 | 4.790511318 |
| 26 | black | lightgreen | 1.448979592 | 0.046399376 | 4.818705831 |
| 27 | blue | magenta | 1.782222222 | 0.047050868 | 4.821507453 |
| 28 | blue | pink | 1.904615385 | 0.047747637 | 4.853290915 |
| 29 | brown | lightgreen | 1.702380952 | 0.039815939 | 4.894752086 |
| 30 | blue | darkorange | 1.706666667 | 0.043284483 | 4.900420518 |
| 31 | purple | yellow | 1.894444444 | 0.034154214 | 4.98204716 |
| 32 | tan | yellow | 1.775 | 0.036576221 | 5.011620783 |
| 33 | darkolivegreen | green | 1.4375 | 0.041312767 | 5.017606519 |
| 34 | darkred | yellow | 1.741666667 | 0.033316613 | 5.034083607 |
| 35 | brown | lightyellow | 1.720238095 | 0.041521928 | 5.052964839 |
| 36 | blue | lightsteelblue1 | 1.32 | 0.037688138 | 5.114465059 |
| 37 | green | purple | 1.819444444 | 0.032547236 | 5.142201133 |
| 38 | green | midnightblue | 1.678571429 | 0.033057968 | 5.171149536 |
| 39 | orange | turquoise | 1.781609195 | 0.037189419 | 5.214911239 |
| 40 | salmon | turquoise | 1.836206897 | 0.039326104 | 5.24130234 |
| 41 | brown | brown4 | 1.5 | 0.034453102 | 5.340879803 |
| 42 | blue | darkslateblue | 1.426666667 | 0.031365581 | 5.450394584 |
| 43 | brown | cyan | 1.796875 | 0.031046054 | 5.465578134 |
| 44 | brown | grey60 | 1.875 | 0.030322277 | 5.483703486 |
| 45 | brown | lightsteelblue1 | 1.416666667 | 0.027923202 | 5.519290494 |
| 46 | blue | brown4 | 1.52 | 0.028656962 | 5.580100226 |
| 47 | greenyellow | royalblue | 1.407407407 | 0.028123542 | 5.642834834 |
| 48 | black | pink | 2 | 0.027527956 | 5.67142999 |
| 49 | lightgreen | purple | 1.650793651 | 0.023680976 | 5.810369046 |
| 50 | midnightblue | purple | 1.619047619 | 0.021213214 | 5.817471449 |
| 51 | darkred | pink | 1.615384615 | 0.0249762 | 5.856582364 |
| 52 | brown | magenta | 1.912037037 | 0.021387913 | 5.87224345 |
| 53 | brown | orange | 1.736111111 | 0.023455713 | 5.883593137 |
| 54 | darkred | purple | 1.759259259 | 0.01873068 | 5.974861032 |
| 55 | blue | lightyellow | 1.874285714 | 0.021213275 | 5.988850172 |
| 56 | brown | darkolivegreen | 1.825 | 0.017341242 | 6.027394196 |
| 57 | greenyellow | lightgreen | 1.507936508 | 0.020425961 | 6.063698137 |
| 58 | orangered4 | turquoise | 1.767241379 | 0.019447337 | 6.080354994 |
| 59 | brown4 | yellow | 1.616666667 | 0.020061633 | 6.11995422 |
| 60 | green | grey60 | 1.839285714 | 0.019946257 | 6.12329944 |
| 61 | black | purple | 1.888888889 | 0.018531694 | 6.174981908 |
| 62 | darkred | grey60 | 1.571428571 | 0.019799316 | 6.187283302 |
| 63 | magenta | purple | 1.666666667 | 0.018401656 | 6.227939137 |
| 64 | grey60 | yellow | 1.971428571 | 0.018262251 | 6.236141882 |
| 65 | brown | darkgrey | 1.847222222 | 0.018772701 | 6.304988127 |
| 66 | steelblue | yellow | 1.8 | 0.018169512 | 6.304995255 |
| 67 | darkred | midnightblue | 1.619047619 | 0.015248253 | 6.318932264 |
| 68 | darkolivegreen | purple | 1.6 | 0.016846288 | 6.328154121 |
| 69 | lightgreen | orangered4 | 1.25 | 0.01721623 | 6.375744751 |
| 70 | brown | sienna3 | 1.697916667 | 0.016983508 | 6.379467123 |
| 71 | darkturquoise | lightgreen | 1.595238095 | 0.017094155 | 6.403008141 |
| 72 | turquoise | yellowgreen | 1.810344828 | 0.015859845 | 6.418675401 |
| 73 | midnightblue | salmon | 1.589285714 | 0.014848484 | 6.430525933 |
| 74 | paleturquoise | yellow | 1.88 | 0.016605 | 6.454701966 |
| 75 | blue | skyblue | 1.966666667 | 0.016376941 | 6.51715275 |
| 76 | red | steelblue | 1.68 | 0.014858089 | 6.585406936 |
| 77 | floralwhite | turquoise | 2.034482759 | 0.014814365 | 6.661821857 |
| 78 | plum1 | yellow | 1.7875 | 0.013937782 | 6.692867911 |
| 79 | cyan | lightgreen | 1.642857143 | 0.012895659 | 6.715588836 |
| 80 | lightgreen | magenta | 1.698412698 | 0.012791589 | 6.751630136 |
| 81 | lightgreen | pink | 1.923076923 | 0.013040205 | 6.765079527 |
| 82 | cyan | darkorange | 1.75 | 0.013255921 | 6.77561558 |
| 83 | brown | skyblue3 | 1.854166667 | 0.012924077 | 6.780613158 |
| 84 | black | orangered4 | 1.625 | 0.0122806 | 6.783427706 |
| 85 | brown | white | 1.840277778 | 0.012908509 | 6.809076347 |
| 86 | magenta | plum1 | 1.527777778 | 0.012925764 | 6.830866081 |
| 87 | blue | plum1 | 1.87 | 0.011910297 | 6.848646959 |
| 88 | darkorange | lightgreen | 1.523809524 | 0.011775665 | 6.898185891 |
| 89 | darkgreen | red | 1.944444444 | 0.01141961 | 6.950249723 |
| 90 | darkslateblue | yellow | 1.816666667 | 0.010738556 | 6.98641613 |
| 91 | darkred | green | 1.90625 | 0.005899799 | 6.990081026 |
| 92 | blue | paleturquoise | 2.032 | 0.011329655 | 7.013232315 |
| 93 | lightcyan1 | red | 1.688888889 | 0.011141726 | 7.029143749 |
| 94 | violet | yellow | 1.95 | 0.010994736 | 7.035949084 |
| 95 | blue | skyblue3 | 1.85 | 0.010489233 | 7.059960648 |
| 96 | darkslateblue | purple | 1.407407407 | 0.010327311 | 7.086469278 |
| 97 | grey60 | midnightblue | 1.87755102 | 0.010111581 | 7.112948831 |
| 98 | darkgreen | lightgreen | 1.619047619 | 0.010303503 | 7.124844425 |
| 99 | darkorange2 | yellow | 1.783333333 | 0.010371901 | 7.140795684 |
| 100 | lightyellow | salmon | 1.767857143 | 0.009781418 | 7.16115104 |
| 101 | orangered4 | yellow | 1.825 | 0.009788623 | 7.163599283 |
| 102 | magenta | orange | 1.722222222 | 0.010011967 | 7.164313942 |
| 103 | darkorange | darkred | 1.666666667 | 0.00909057 | 7.167835258 |
| 104 | black | orange | 1.880952381 | 0.009328396 | 7.173260466 |
| 105 | darkslateblue | midnightblue | 1.380952381 | 0.009742301 | 7.186257131 |
| 106 | lightsteelblue1 | purple | 1.666666667 | 0.008839992 | 7.202222488 |
| 107 | red | white | 1.844444444 | 0.009597585 | 7.212611763 |
| 108 | darkorange2 | red | 1.644444444 | 0.009457332 | 7.26532999 |
| 109 | darkolivegreen | midnightblue | 1.742857143 | 0.008134599 | 7.292784929 |
| 110 | red | violet | 1.816666667 | 0.008776643 | 7.358036792 |
| 111 | cyan | darkturquoise | 1.8125 | 0.008823388 | 7.368320636 |
| 112 | darkorange | magenta | 1.814814815 | 0.008334577 | 7.43788565 |
| 113 | darkgreen | orangered4 | 1.541666667 | 0.008268142 | 7.474747491 |
| 114 | black | brown | 1.964285714 | 0.004215902 | 7.474905315 |
| 115 | lightyellow | tan | 1.875 | 0.007825751 | 7.484645284 |
| 116 | cyan | lightcyan1 | 1.5 | 0.007861569 | 7.552869723 |
| 117 | darkgreen | greenyellow | 1.87037037 | 0.007191836 | 7.60255343 |
| 118 | lightgreen | orange | 1.714285714 | 0.006945885 | 7.628806707 |
| 119 | darkturquoise | orange | 1.666666667 | 0.007310715 | 7.630314957 |
| 120 | orangered4 | violet | 1.3125 | 0.007320206 | 7.672878468 |
| 121 | cyan | white | 1.75 | 0.007079662 | 7.704471553 |
| 122 | black | lightsteelblue1 | 1.714285714 | 0.007153607 | 7.706183461 |
| 123 | lightyellow | midnightblue | 1.836734694 | 0.006666445 | 7.734551632 |
| 124 | darkgreen | violet | 1.791666667 | 0.006885855 | 7.74124216 |
| 125 | greenyellow | orangered4 | 1.777777778 | 0.006358871 | 7.781086197 |
| 126 | lightgreen | skyblue3 | 1.607142857 | 0.006407591 | 7.854474503 |
| 127 | royalblue | skyblue | 2.138888889 | 0.006417543 | 7.858269038 |
| 128 | darkorange | tan | 1.895833333 | 0.005205656 | 7.899654828 |
| 129 | lightgreen | white | 1.714285714 | 0.006156394 | 7.910545945 |
| 130 | darkred | lightgreen | 1.785714286 | 0.005267578 | 7.920285458 |
| 131 | greenyellow | lightyellow | 1.888888889 | 0.005869522 | 7.950227106 |
| 132 | darkorange | orange | 1.833333333 | 0.005736858 | 8.007411936 |
| 133 | cyan | darkgreen | 1.979166667 | 0.00575551 | 8.009407886 |
| 134 | bisque4 | red | 1.733333333 | 0.005616162 | 8.013434506 |
| 135 | darkred | tan | 1.979166667 | 0.002875039 | 8.027165103 |
| 136 | darkgreen | lightcyan1 | 1.555555556 | 0.005630676 | 8.039540457 |
| 137 | greenyellow | lightcyan1 | 1.592592593 | 0.005406169 | 8.078534128 |
| 138 | plum1 | steelblue | 1.65 | 0.00546978 | 8.099264055 |
| 139 | darkolivegreen | lightsteelblue1 | 1.466666667 | 0.004987117 | 8.139780182 |
| 140 | lightgreen | lightsteelblue1 | 1.571428571 | 0.004975833 | 8.163434534 |
| 141 | darkolivegreen | grey60 | 1.857142857 | 0.004776652 | 8.188070093 |
| 142 | lightcyan1 | lightgreen | 1.571428571 | 0.004442515 | 8.295145058 |
| 143 | black | lightcyan1 | 1.738095238 | 0.004371844 | 8.309892234 |
| 144 | brown4 | tan | 1.833333333 | 0.004473391 | 8.317977309 |
| 145 | midnightblue | steelblue | 1.885714286 | 0.004581575 | 8.335230972 |
| 146 | royalblue | steelblue | 1.733333333 | 0.004553221 | 8.36272514 |
| 147 | darkorange | yellow | 2.041666667 | 0.002270613 | 8.367665264 |
| 148 | darkolivegreen | yellow | 1.98 | 0.002249535 | 8.381119922 |
| 149 | lightgreen | turquoise | 1.901477833 | 0.002221602 | 8.399146081 |
| 150 | darkred | lightsteelblue1 | 1.611111111 | 0.003422875 | 8.422230359 |
| 151 | darkgreen | skyblue3 | 1.791666667 | 0.004173913 | 8.48934619 |
| 152 | greenyellow | violet | 1.861111111 | 0.00396562 | 8.512362713 |
| 153 | lightsteelblue1 | yellow | 2 | 0.0019979 | 8.552262766 |
| 154 | greenyellow | red | 1.844444444 | 0.001977615 | 8.566984905 |
| 155 | purple | tan | 2.305555556 | 0.001929716 | 8.60235855 |
| 156 | brown4 | darkred | 1.611111111 | 0.003454468 | 8.607445465 |
| 157 | lightsteelblue1 | pink | 1.820512821 | 0.00362845 | 8.691393523 |
| 158 | brown | darkslateblue | 1.958333333 | 0.001801365 | 8.701656483 |
| 159 | blue | orange | 1.933333333 | 0.001800449 | 8.702390168 |
| 160 | green | tan | 2.234375 | 0.001786985 | 8.713219335 |
| 161 | lightcyan1 | violet | 1.666666667 | 0.003571301 | 8.714297157 |
| 162 | ivory | lightcyan1 | 1.444444444 | 0.003558623 | 8.719427869 |
| 163 | darkred | salmon | 1.895833333 | 0.001715635 | 8.772004104 |
| 164 | darkorange2 | steelblue | 1.666666667 | 0.003401414 | 8.784612319 |
| 165 | brown4 | midnightblue | 1.714285714 | 0.002978882 | 8.816747346 |
| 166 | green | lightsteelblue1 | 1.875 | 0.001618601 | 8.855999237 |
| 167 | darkorange | white | 1.944444444 | 0.00319926 | 8.873008364 |
| 168 | lightyellow | yellow | 1.935714286 | 0.001594902 | 8.877279031 |
| 169 | bisque4 | lightgreen | 1.666666667 | 0.002910041 | 9.009707397 |
| 170 | sienna3 | yellowgreen | 1.625 | 0.00283631 | 9.046731808 |
| 171 | darkorange | plum1 | 1.875 | 0.002774788 | 9.078369082 |
| 172 | green | salmon | 2.171875 | 0.001362098 | 9.104915912 |
| 173 | darkorange | purple | 2.018518519 | 0.001320098 | 9.150101882 |
| 174 | darkturquoise | floralwhite | 1.777777778 | 0.002522768 | 9.215739212 |
| 175 | floralwhite | paleturquoise | 1.666666667 | 0.00245002 | 9.257952976 |
| 176 | salmon | tan | 2.265625 | 0.001222057 | 9.261434668 |
| 177 | purple | salmon | 2.152777778 | 0.001178081 | 9.31430764 |
| 178 | lightsteelblue1 | royalblue | 1.722222222 | 0.002350834 | 9.317574062 |
| 179 | darkturquoise | lightcyan1 | 1.833333333 | 0.002065906 | 9.503972229 |
| 180 | midnightblue | tan | 2.196428571 | 0.001006579 | 9.541286476 |
| 181 | darkturquoise | turquoise | 2.074712644 | 0.000982795 | 9.575784531 |
| 182 | royalblue | turquoise | 2.091954023 | 0.000981343 | 9.577917644 |
| 183 | darkolivegreen | darkred | 1.833333333 | 0.000973983 | 9.588777584 |
| 184 | bisque4 | yellowgreen | 1.666666667 | 0.001946727 | 9.589696387 |
| 185 | black | magenta | 2.071428571 | 0.000972282 | 9.591300351 |
| 186 | blue | cyan | 2.07 | 0.000966567 | 9.599804923 |
| 187 | blue | sienna3 | 1.92 | 0.000879433 | 9.736101576 |
| 188 | greenyellow | yellow | 2.061111111 | 0.000838121 | 9.805516517 |
| 189 | lightcyan1 | orangered4 | 1.666666667 | 0.001485318 | 9.979974624 |
| 190 | darkslateblue | green | 1.958333333 | 0.000729305 | 10.0061531 |
| 191 | brown | plum1 | 1.979166667 | 0.000687792 | 10.09070215 |
| 192 | grey60 | purple | 2.095238095 | 0.000666346 | 10.13640294 |
| 193 | lightsteelblue1 | midnightblue | 1.904761905 | 0.000662997 | 10.14367223 |
| 194 | red | royalblue | 2.1 | 0.000596914 | 10.29515147 |
| 195 | lightgreen | red | 1.914285714 | 0.000588751 | 10.31501831 |
| 196 | cyan | greenyellow | 1.944444444 | 0.000585927 | 10.32195326 |
| 197 | blue | orangered4 | 2.04 | 0.00054214 | 10.43401032 |
| 198 | black | darkturquoise | 2.130952381 | 0.000532918 | 10.45876248 |
| 199 | blue | steelblue | 1.984 | 0.000499094 | 10.55336269 |
| 200 | black | greenyellow | 2.166666667 | 0.000486815 | 10.5893006 |
| 201 | magenta | yellow | 2.105555556 | 0.000467601 | 10.64739767 |
| 202 | brown | pink | 2.131410256 | 0.000467321 | 10.64826213 |
| 203 | darkorange | green | 2.333333333 | 0.000425397 | 10.7838643 |
| 204 | brown | darkturquoise | 1.979166667 | 0.00041889 | 10.80610255 |
| 205 | brown4 | purple | 1.851851852 | 0.000411872 | 10.83048041 |
| 206 | lightsteelblue1 | tan | 2.166666667 | 0.000384807 | 10.9285409 |
| 207 | brown4 | green | 2.1875 | 0.000382568 | 10.93695783 |
| 208 | blue | royalblue | 2.22 | 0.000302358 | 11.27641519 |
| 209 | black | cyan | 2.098214286 | 0.000279892 | 11.3878039 |
| 210 | brown | greenyellow | 2.069444444 | 0.000269482 | 11.44248729 |
| 211 | saddlebrown | turquoise | 2.027586207 | 0.000265724 | 11.46274614 |
| 212 | darkred | darkslateblue | 1.888888889 | 0.00025693 | 11.51130207 |
| 213 | brown | orangered4 | 2.0625 | 0.000256027 | 11.51637755 |
| 214 | black | darkred | 2.05952381 | 0.000249449 | 11.55392725 |
| 215 | orangered4 | red | 2.1 | 0.000246628 | 11.57033552 |
| 216 | darkorange | midnightblue | 2.166666667 | 0.000234897 | 11.64064742 |
| 217 | darkorange | lightsteelblue1 | 2 | 0.000234842 | 11.64098207 |
| 218 | green | lightgreen | 2.0625 | 0.000199808 | 11.87406238 |
| 219 | sienna3 | yellow | 2.1 | 0.000197804 | 11.88860339 |
| 220 | blue | lightcyan1 | 2.16 | 0.000183187 | 11.99935431 |
| 221 | green | skyblue3 | 1.96875 | 0.000181828 | 12.01010307 |
| 222 | darkorange | salmon | 2.3125 | 0.000160941 | 12.18614633 |
| 223 | orange | purple | 2.166666667 | 0.000151015 | 12.27798133 |
| 224 | black | darkgreen | 2.154761905 | 0.000148199 | 12.30513527 |
| 225 | plum1 | purple | 1.972222222 | 0.000146767 | 12.31915018 |
| 226 | orange | salmon | 1.916666667 | 0.000145269 | 12.33394711 |
| 227 | lightsteelblue1 | salmon | 2.125 | 0.000142062 | 12.36615322 |
| 228 | lightgreen | royalblue | 2 | 0.00013515 | 12.43811778 |
| 229 | brown | royalblue | 2.263888889 | 0.000122691 | 12.57764397 |
| 230 | blue | darkorange2 | 2.24 | 0.000120777 | 12.60033132 |
| 231 | lightgreen | midnightblue | 2 | 0.000115631 | 12.66314603 |
| 232 | lightgreen | violet | 1.964285714 | 0.000113327 | 12.69218817 |
| 233 | brown4 | salmon | 2.125 | 0.000112236 | 12.70614483 |
| 234 | orangered4 | purple | 2.055555556 | 0.000111961 | 12.70968087 |
| 235 | brown | lightcyan1 | 2.125 | 9.27739E-05 | 12.98088442 |
| 236 | magenta | midnightblue | 1.920634921 | 8.77141E-05 | 13.06179485 |
| 237 | blue | yellowgreen | 2.13 | 8.68113E-05 | 13.07672004 |
| 238 | darkred | orange | 1.972222222 | 8.39534E-05 | 13.12501449 |
| 239 | brown | yellowgreen | 2.052083333 | 8.27156E-05 | 13.14644323 |
| 240 | darkred | magenta | 2 | 7.93896E-05 | 13.2056531 |
| 241 | darkolivegreen | darkslateblue | 1.933333333 | 7.79373E-05 | 13.23228844 |
| 242 | darkturquoise | red | 2.033333333 | 7.69414E-05 | 13.25084236 |
| 243 | sienna3 | tan | 2.3125 | 7.49189E-05 | 13.28927345 |
| 244 | darkolivegreen | lightgreen | 2.028571429 | 7.30479E-05 | 13.32575996 |
| 245 | midnightblue | plum1 | 2 | 6.24113E-05 | 13.55279574 |
| 246 | pink | purple | 2.102564103 | 6.23986E-05 | 13.55308905 |
| 247 | lightyellow | royalblue | 1.880952381 | 6.03166E-05 | 13.60204673 |
| 248 | brown | darkorange2 | 2.027777778 | 5.58319E-05 | 13.71351289 |
| 249 | purple | sienna3 | 2.277777778 | 5.5511E-05 | 13.7218298 |
| 250 | bisque4 | greenyellow | 1.740740741 | 5.54665E-05 | 13.72298668 |
| 251 | brown4 | darkorange | 2 | 5.11831E-05 | 13.83893596 |
| 252 | darkred | sienna3 | 2.208333333 | 5.09032E-05 | 13.8468453 |
| 253 | grey60 | pink | 2.043956044 | 4.61975E-05 | 13.98678843 |
| 254 | salmon | yellowgreen | 1.84375 | 3.93564E-05 | 14.21800512 |
| 255 | darkorange | sienna3 | 2.208333333 | 3.7984E-05 | 14.26921071 |
| 256 | darkolivegreen | tan | 2.25 | 2.93858E-05 | 14.63948416 |
| 257 | darkgrey | greenyellow | 2.074074074 | 2.60894E-05 | 14.81113748 |
| 258 | darkturquoise | magenta | 2.166666667 | 2.53175E-05 | 14.85446834 |
| 259 | darkorange | greenyellow | 2.111111111 | 2.26116E-05 | 15.017542 |
| 260 | brown4 | lightsteelblue1 | 1.777777778 | 1.71983E-05 | 15.41234013 |
| 261 | darkslateblue | salmon | 2.208333333 | 1.34483E-05 | 15.76717388 |
| 262 | black | darkorange | 2.297619048 | 7.39956E-06 | 16.62909215 |
| 263 | cyan | darkred | 2.0625 | 5.40599E-06 | 17.0819724 |
| 264 | orange | orangered4 | 2.208333333 | 5.2314E-06 | 17.12933388 |
| 265 | black | royalblue | 2.511904762 | 3.49711E-06 | 17.71036637 |
| 266 | cyan | orange | 2.020833333 | 3.27317E-06 | 17.80584463 |

Supplement table 4 Correspondence between IMCC (determined by the formula 1) and inter-module average shortest path (also known as inter-module average characteristic path length).

Supplement table 5

| Group | Relative frequency (%)  In (-∞,5.2] | Relative frequency (%)  in (5.2,7.3] | Relative frequency (%)  in (7.3,10.2] | Relative frequency (%)  in (10.2,+∞) | P-value | Distance in PCA |
| --- | --- | --- | --- | --- | --- | --- |
| Vehicle | 18.05 | 28.95 | 23.68 | 29.32 | — | — |
| BA | 11.11 | 21.30 | 40.74 | 26.85 | 0.0004614 | 20.09 |
| JA | 44.87 | 39.74 | 14.10 | 1.28 | 1.971e-09 | 41.40 |
| UA | 23.81 | 13.10 | 23.81 | 39.29 | 0.02365 | 19.59 |
| CM | 30.68 | 25.00 | 23.86 | 20.45 | 0.06374 | 15.94 |

Supplement table 5 Distribution of IMCC score, statistical significance and distance in PCA. IMCC score were divided into 4 intervals by quartiles for contingency table chi-square test, with relative frequency in each interval shown in table. Statistical significance and distance in PCA of treated-groups compared with vehicle were also shown.

Supplement table 6

|  | Module | Betweenness |
| --- | --- | --- |
| Vehicle | blue | 0.1667883 |
|  | brown | 0.16076895 |
|  | lightgreen | 0.08671703 |
|  | turquoise | 0.06267317 |
|  | yellow | 0.06091004 |
| BA | turquoise | 0.01085945 |
|  | green | 0.01041516 |
|  | salmon | 0.01010595 |
| JA | turquoise | 0.28994411 |
|  | magenta | 0.15836253 |
|  | blue | 0.1521021 |
|  | tan | 0.15165165 |
|  | darkorange | 0.13001752 |
| UA | turquoise | 0.03001522 |
|  | brown | 0.03001522 |

Supplement table 6 Identified connectors by betweenness.

Supplement table 7

|  | **Origin characteristic path length** | **Altered characteristic path length** | **Variation of characteristic path length** | **Δs** | **Origin density** | **Altered density** | **Variation of density** | **Δt** |
| --- | --- | --- | --- | --- | --- | --- | --- | --- |
| Vehicle-brown | 1.848 | 1.918 | 0.07 | 0.037878788 | 0.271 | 0.247 | -0.024 | -0.088560886 |
| Vehicle-blue | 1.848 | 1.926 | 0.078 | 0.042207792 | 0.271 | 0.248 | -0.023 | -0.084870849 |
| Vehicle-salmon | 1.848 | 1.863 | 0.015 | 0.008116883 | 0.271 | 0.266 | -0.005 | -0.018450185 |
| Vehicle-greenyellow | 1.848 | 1.859 | 0.011 | 0.005952381 | 0.271 | 0.267 | -0.004 | -0.014760148 |
| Vehicle-orange | 1.848 | 1.854 | 0.006 | 0.003246753 | 0.271 | 0.27 | -0.001 | -0.003690037 |
| Vehicle-red | 1.848 | 1.857 | 0.009 | 0.00487013 | 0.271 | 0.27 | -0.001 | -0.003690037 |
| Vehicle-orangered4 | 1.848 | 1.855 | 0.007 | 0.003787879 | 0.271 | 0.27 | -0.001 | -0.003690037 |
| Vehicle-darkolivegreen | 1.848 | 1.851 | 0.003 | 0.001623377 | 0.271 | 0.271 | 0 | 0 |
| Vehicle-cyan | 1.848 | 1.853 | 0.005 | 0.002705628 | 0.271 | 0.271 | 0 | 0 |
| Vehicle-magenta | 1.848 | 1.852 | 0.004 | 0.002164502 | 0.271 | 0.271 | 0 | 0 |
| Vehicle-brown4 | 1.848 | 1.849 | 0.001 | 0.000541126 | 0.271 | 0.272 | 0.001 | 0.003690037 |
| Vehicle-royalblue | 1.848 | 1.858 | 0.01 | 0.005411255 | 0.271 | 0.272 | 0.001 | 0.003690037 |
| Vehicle-darkturquoise | 1.848 | 1.858 | 0.01 | 0.005411255 | 0.271 | 0.273 | 0.002 | 0.007380074 |
| JA-pink | 2.865 | 2.939 | 0.074 | 0.02582897 | 0.101 | 0.099 | -0.002 | -0.01980198 |
| JA-magenta | 2.865 | 3.073 | 0.208 | 0.072600349 | 0.101 | 0.099 | -0.002 | -0.01980198 |
| JA-royalblue | 2.865 | 2.961 | 0.096 | 0.033507853 | 0.101 | 0.1 | -0.001 | -0.00990099 |
| JA-salmon | 2.865 | 2.94 | 0.075 | 0.02617801 | 0.101 | 0.1 | -0.001 | -0.00990099 |
| JA-greenyellow | 2.865 | 2.951 | 0.086 | 0.030017452 | 0.101 | 0.1 | -0.001 | -0.00990099 |
| JA-sienna3 | 2.865 | 2.982 | 0.117 | 0.040837696 | 0.101 | 0.101 | 0 | 0 |
| JA-lightcyan | 2.865 | 2.874 | 0.009 | 0.003141361 | 0.101 | 0.101 | 0 | 0 |
| JA-lightcyan1 | 2.865 | 2.87 | 0.005 | 0.001745201 | 0.101 | 0.101 | 0 | 0 |
| JA-lightgreen | 2.865 | 2.877 | 0.012 | 0.004188482 | 0.101 | 0.101 | 0 | 0 |
| JA-red | 2.865 | 2.87 | 0.005 | 0.001745201 | 0.101 | 0.103 | 0.002 | 0.01980198 |
| JA-saddlebrown | 2.865 | 2.877 | 0.012 | 0.004188482 | 0.101 | 0.103 | 0.002 | 0.01980198 |
| JA-mediumpurple3 | 2.865 | 2.889 | 0.024 | 0.008376963 | 0.101 | 0.103 | 0.002 | 0.01980198 |
| JA-lightsteelblue1 | 2.865 | 2.877 | 0.012 | 0.004188482 | 0.101 | 0.104 | 0.003 | 0.02970297 |
| JA-steelblue | 2.865 | 2.895 | 0.03 | 0.010471204 | 0.101 | 0.104 | 0.003 | 0.02970297 |
| JA-white | 2.865 | 2.868 | 0.003 | 0.00104712 | 0.101 | 0.105 | 0.004 | 0.03960396 |
| JA-darkred | 2.865 | 2.868 | 0.003 | 0.00104712 | 0.101 | 0.105 | 0.004 | 0.03960396 |

Supplement table 7 Connectors detected by VRCD.
