# Supplementary figures and images for: IMCC: A Novel Quantitative Approach Revealing Variation of Global Modular Map and Local Inter-Module Coordination Among Differential Drug’s Targeted Cerebral Ischemic Networks
Source: Front Pharmacol. 2021 Apr 16;12:637253. doi: 10.3389/fphar.2021.637253 (PMC8087074; doi:10.3389/fphar.2021.637253)

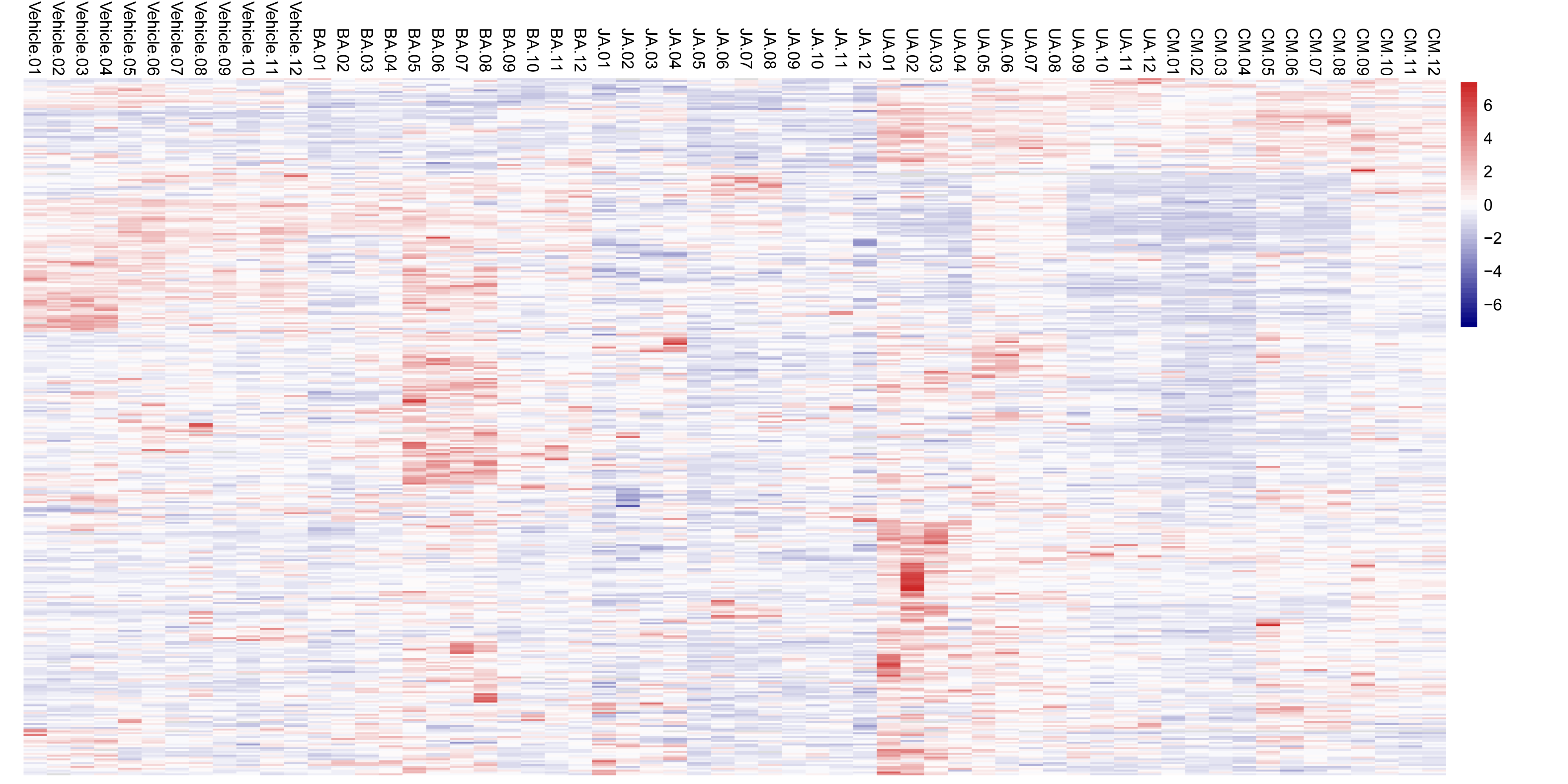

Supplement: Supplementary file 1 [file datasheet1.zip › Supplementary figure 1-3/Supplementary figure 1.png]

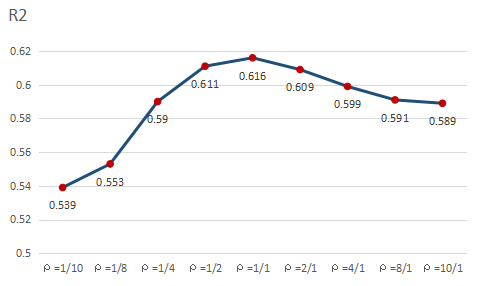

Supplement: Supplementary file 1 [file datasheet1.zip › Supplementary figure 1-3/Supplementary figure 2.png]

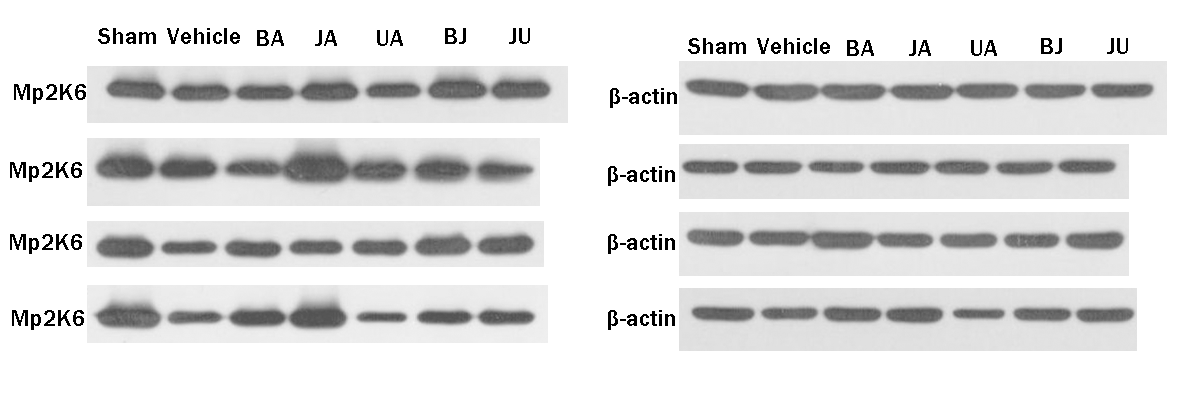

Supplement: Supplementary file 1 [file datasheet1.zip › Supplementary figure 1-3/supplementary figure 3.png]
